# Rapid reconstitution of ubiquitinated nucleosome using a non-denatured histone octamer ubiquitylation approach

**DOI:** 10.1186/s13578-024-01265-x

**Published:** 2024-06-17

**Authors:** Weijie Li, Peirong Cao, Pengqi Xu, Fahui Sun, Chi Wang, Jiale Zhang, Shuqi Dong, Jon R. Wilson, Difei Xu, Hengxin Fan, Zhenhuan Feng, Xiaofei Zhang, Qingjun Zhu, Yingzhi Fan, Nick Brown, Neil Justin, Steven J Gamblin, He Li, Ying Zhang, Jun He

**Affiliations:** 1 Tomas Lindahl Nobel Laureate Laboratory, The Seventh Affiliated Hospital, Sun Yat-Sen University, Shenzhen, 518107 China; 2 CAS Key Laboratory of Regenerative Biology, Guangdong Provincial Key Laboratory of Stem Cell and Regenerative Medicine, GIBH-HKU Guangdong-Hong Kong Stem Cell and Regenerative Medicine Research Centre, GIBH-CUHK Joint Research Laboratory on Stem Cell and Regenerative Medicine, Guangzhou Institutes of Biomedicine and Health, Chinese Academy of Sciences, Guangzhou, 510530 China; 3 Key Laboratory of Biological Targeting Diagnosis, Therapy and Rehabilitation of Guangdong Higher Education Institutes, The Fifth Affiliated Hospital of Guangzhou Medical University, Guangzhou, 510799 China; 4 School of Life Sciences, University of Science and Technology of China, Hefei, 230026 China; 5 Department of Neuroscience, City University of Hong Kong, Kowloon Tong, Hong Kong SAR P.R. China; 6 University of Chinese Academy of Sciences, Beijing, 100049 China; 7 Francis Crick Institute, 1 Midland Road, London, NW1 1AT UK; 8 School of Chinese Medicine, Hong Kong Baptist University, Hong Kong, China

**Keywords:** Non-denatured, Ubiquitinated histone octamer, Ubiquitin-like, Nucleosomes

## Abstract

**Background:**

Histone ubiquitination modification is emerging as a critical epigenetic mechanism involved in a range of biological processes. In vitro reconstitution of ubiquitinated nucleosomes is pivotal for elucidating the influence of histone ubiquitination on chromatin dynamics.

**Results:**

In this study, we introduce a Non-Denatured Histone Octamer Ubiquitylation (NDHOU) approach for generating ubiquitin or ubiquitin-like modified histone octamers. The method entails the co-expression and purification of histone octamers, followed by their chemical cross-linking to ubiquitin using 1,3-dibromoacetone. We demonstrate that nucleosomes reconstituted with these octamers display a high degree of homogeneity, rendering them highly compatible with in vitro biochemical assays. These ubiquitinated nucleosomes mimic physiological substrates in function and structure. Additionally, we have extended this method to cross-linking various histone octamers and three types of ubiquitin-like proteins.

**Conclusions:**

Overall, our findings offer an efficient strategy for producing ubiquitinated nucleosomes, advancing biochemical and biophysical studies in the field of chromatin biology.

**Supplementary Information:**

The online version contains supplementary material available at 10.1186/s13578-024-01265-x.

## Introduction

Eukaryotic genomes undergo packaging within a nucleoprotein complex known as chromatin. Histone post-translational modifications (PTMs) are shown to have a crucial role in epigenetic regulation [1–3]. Especially, ubiquitylation of histone tails on specific sites, has been linked to fundamental biological processes including chromatin dynamics, transcriptional regulation, genome stability and many diseases [4–7]. Specifically, ubiquitylation of histone H2A Lys15 (H2AK15Ub) mediates recruitment of p53-binding protein (53BP1) to signal DNA damage repair [8]. Ubiquitylation of H2BK120 modulates the interaction between adjacent nucleosomes, hence altering higher-order chromosome structure [9]. The exact processes through which chromatin-regulating enzymes recognize and respond to ubiquitinated nucleosomes are not comprehensively understood. A detailed elucidation of these ubiquitin-mediated pathways is essential, not only for advancing the fundamental knowledge but also for informing the development of novel therapeutic interventions [4, 10]. The required rigorous structural and functional investigations typically involve a suite of in vitro methodologies, which depend on the accessibility of site-specific ubiquitinated nucleosomes characterized by a high degree of uniformity.

Ubiquitinated nucleosomes are typically obtained by endogenous extraction from mammalian cell culture, including HeLa cells and calf thymus cells [11, 12], or by in vitro biochemical reconstitution from purified histones [13, 14]. However, nucleosomes derived from endogenous sources present significant heterogeneity in their PTMs [11, 12], which poses challenges for site-specific investigations. In contrast, nucleosomes that are biochemically reconstituted offer the advantage of homogeneity, facilitating the generation of high yields that are amenable to detailed study. In the standard reconstitution of ubiquitinated nucleosomes, the four core histones (H2A, H2B, H3, and H4) are initially isolated from various inclusion bodies under denaturing conditions [13, 15]. To ubiquitinate H2A or H2B, the conventional process involving chemical synthesis, purification, dialysis, and lyophilization were performed under denaturing conditions, which facilitate the conjugation of ubiquitin to the histones [8, 14, 16]. Subsequently, the ubiquitinated histone variant, along with other histones, is refolded into soluble histone octamers, which can be then reconstituted into nucleosomes *via* a two-day dialysis [13]. The complexity of this protocol underscores the challenges inherent in obtaining high yield ubiquitinated nucleosomes. Additionally, the attachment of ubiquitin to the individual denatured histone may lead to steric hindrance, which can adversely affect octamer assembly and further reduce the yield of properly reconstituted nucleosomes.

In vivo, histone ubiquitylation predominantly occurs on intact histone octamers or nucleosomes [5, 10, 17]. Consequently, it is logical to ubiquitinate soluble histone octamers or nucleosomes directly, a process that may be favored to circumvent steric hindrance, thereby streamlining the procedure and potentially increasing yield. Indeed, a previously described method, which employs the co-expression and co-purification of histone octamers from a single polycistronic vector, has successfully synthesized recombinant histone octamers in a soluble form, markedly reducing processing time and enhancing yield [18]. Nonetheless, it remains unknown whether ubiquitination can be efficiently carried out on histone octamers in a soluble state in vitro. Moreover, in the context of histone ubiquitylation, the specificity of the reaction typically targets the ε-amino of lysine residues on histones and the G76 position on ubiquitin to form isopeptide bonds [19, 20]. To facilitate this reaction in vitro, chemical approaches have been devised that utilize the sulfhydryl group of cysteine, employing hydrazide-, disulfide-, or 1,3-dichloroacetone (DCA)-derived isopeptide bond analogs [16, 20–33]. While disulfide-based ubiquitination modifications are unstable under reducing conditions, DCA-mediated ubiquitination of histones is stable, resistant to deubiquitination enzymes, and favorable for structural studies due to the bond’s close resemblance to native isopeptide bonds. Recent studies have indicated that using the more electrophilic 1,3-dibromoacetone (DBA) from the same main group in the periodic table as the cross-linking reagent significantly improves the yield of modified histones when compared to DCA [16, 26]. Nevertheless, the applicability of DCA/DBA-mediated chemical cross-linking directly to histone octamers under soluble condition has not yet been established.

**Fig. 1:**
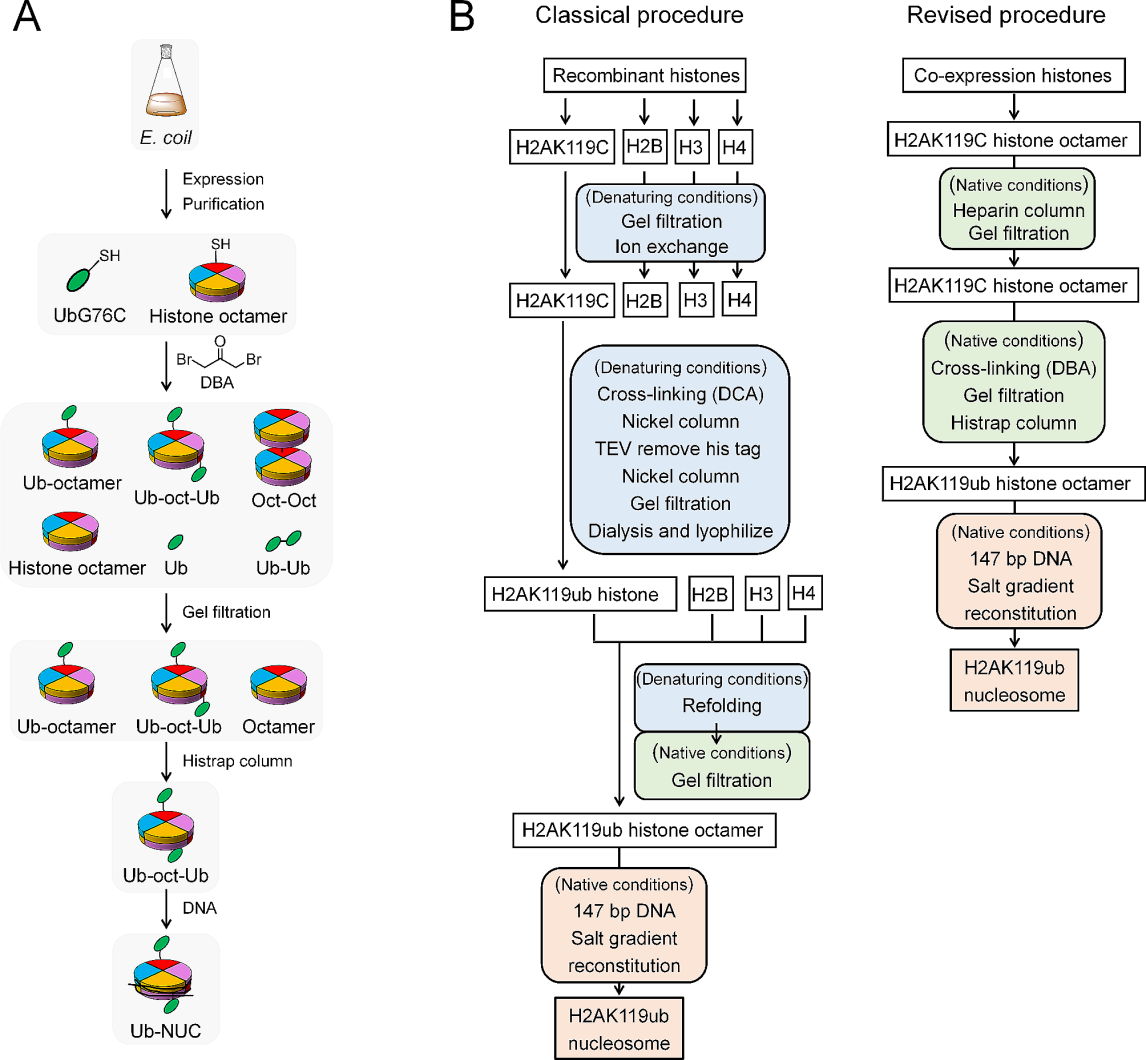
Schematic for the **A** DBA-mediated chemical modification of histone octamers for obtaining ubiquitinated nucleosomes and **B** comparison with previous methods, including the expression and purification of histone octamer mutants and ubiquitin (G76C), DBA-mediated chemical modification of histone octamers and further purification by two standard chromatography steps, and the reconstitution of ubiquitinated nucleosomes in vitro

**Fig. 2:**
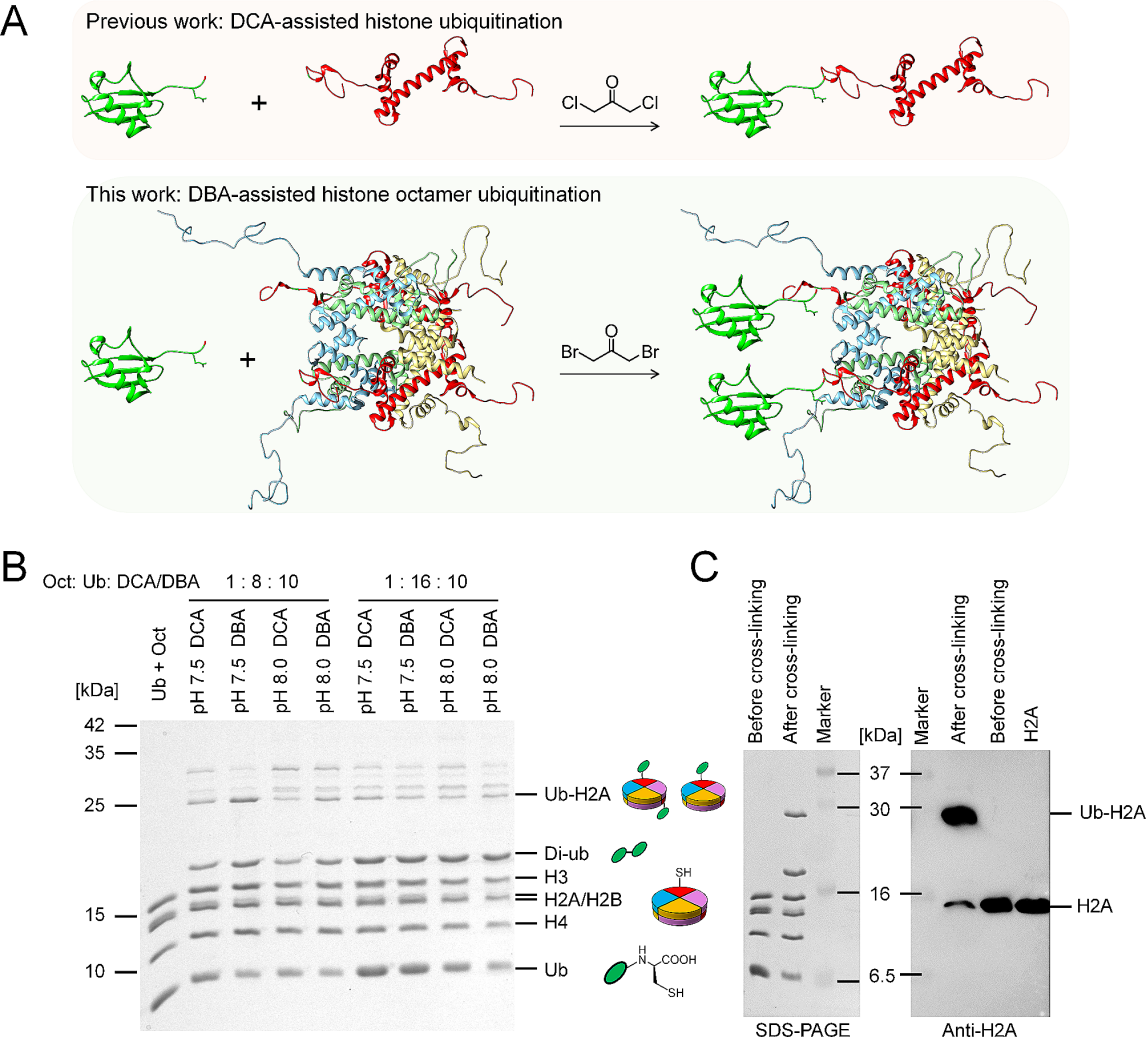
DCA/DBA-mediated synthesis of H2AK119 ubiquitinated histone octamer. **A** Schematics for prior study involving DCA-mediated histone ubiquitination and this work involving DBA-mediated histone octamer ubiquitination; **B** Optimization of the reaction’s efficiency and yield is achievable by adjusting various parameters, as evidenced by the results obtained from SDS-PAGE analysis; **C** SDS-PAGE and Western blotting analysis of the optimized cross-linking between ubiquitin and H2AK119 on the octamer

As an alternative to above existing approaches, we raised the hypothesis that it would be possible to conjugate G76 of ubiquitin to specific lysine sites on H2A/H2B tails on histone octamer form. This approach exploits the latent reactivity of cysteine side chains to chemically generate histone octamer with site-specific ubiquitination and aims to preserve the structural and functional characteristics that are analogous to the classically occurring form (Figs. 1 and 2A). Moreover, this scheme provides a simple and affordable route to large quantities of specifically ubiquitinated octamers. It is also versatile enough to permit modifications at various histone sites and can be further adapted to introduce other ubiquitin-like modifications, such as SUMOylation, NEDDylation, and UFMylation. The ubiquitinated histone octamer mimics produced are stable under reducing conditions and display a bond length that differs by only one bond from the native isopeptide bond. We further proved this strategy is compatible with the in vitro nucleosome reconstitution, resulting in high yield of ubiquitinated nucleosomes that function similarly to their classical counterparts and are useful for the study of histone-ubiquitination’s influence on chromatin structure and function. Therefore, they are well-suited for use in biochemical and biophysical studies of chromatin.

## Methods

### Expression and purification of Histone Octamers

The resulting plasmid encoding all four core histones (including unmodified and mutated histone octamers) was transformed into Rosetta (DE3) cells and plated onto an LB agar plate containing kanamycin (50 µg/mL) and chloramphenicol (34 µg/mL). The plate was incubated overnight (∼ 16 h) at 37 °C. For a 1 L scale preparation, one colony was inoculated into 10 mL LB medium containing kanamycin (50 µg/mL) and chloramphenicol (34 µg/mL). This starter culture was shaken at 220 rpm at 37 °C until slightly cloudy. Subsequently, the culture was amplified into 1 L of the TB medium and was grown for another 4 ∼ 5 h at 37 °C. When the OD_600_ reaches 0.4 ∼ 0.5, histones co-expression was induced by adding 0.4 mM IPTG (Isopropyl β-D-Thiogalactoside). The culture was further shaken at 180 rpm at 37 °C for overnight (∼ 18 h). Cells were harvested by centrifugation at 4000 rpm for 30 min at 4 °C. Cell pellets were processed immediately or stored at -80 °C for future purification.

Cell pellets were resuspended in lysis buffer (20 mM Tris-HCl, pH 8.0, 500 mM NaCl, 0.1 mM Na-EDTA, 0.5 mM PMSF, 10 mM β-ME), and the resuspended cells were lysed by Ultra highly pressure homogenizer (JN-Mini Pro) and clarified by centrifugation. The supernatant was collected and the clarified lysate was then loaded onto a 5 mL heparin column (GE Healthcare). And then washed by a 20 column volumes (CV) buffer A (20 mM Tris-HCl, pH 8.0, 500 mM NaCl, 0.1 mM Na-EDTA, 10 mM β-ME). The bound proteins were eluted by increasing the NaCl concentration from 500 mM to 2 M linearly over 24 CV at 2 mL/min. Each fraction was analyzed by SDS-PAGE, and the purified histone octamer eluted from heparin column were then concentrated up to ∼ 10 mg/mL. The concentrated sample was then injected onto a Superdex 200 16/600 column (2 M NaCl, 1 x PBS, pH 7.5). The histone octamer peak was eluted at an elution volume of ∼ 64 mL. The peak fractions were pooled according to SDS-PAGE analysis and concentrated up to 10 mg/mL, aliquoted and flash-frozen in liquid nitrogen and stored in the refrigerator at -80^o^C for long-term storage.

### Expression and purification of Ubiquitin, SUMO3, UFM1 and NEDD8

The pGEX 6P-1 plasmid vector was engineered to express GST fusion protein, followed by the human rhinovirus 3 C protease (3 C protease) cleavage site, a hexahistidine tag, and the target protein (Ubiquitin, SUMO3, UFM1 and NEDD8) immediately downstream of the hexahistidine tag. The expression and purification process of ubiquitin (G76C) was briefly described as follows. The plasmid containing the ubiquitin mutant gene was transformed into *E. coli* BL21 (DE3) cells. The cells were grown in an LB medium containing 50 µg/mL kanamycin to OD_600_ of 1.5 ∼ 2.0 and then induced by 0.4 mM IPTG overnight at 20 °C. After harvesting the induced cells, and then the cells were resuspended in lysis buffer (1 x PBS, pH 7.5, 1 mM PMSF, 5 mM DTT) and lysed by Ultra high-pressure homogenizer. After centrifugation at 22 000 rpm for 60 min at 4 °C. The supernatant was loaded onto a 10 mL GST resin pre-equilibrated in the GST buffer (1 x PBS, pH 7.5, 5 mM DTT), and further washed 20 column volumes (CV) by GST buffer. Then the 3 C-GST was loaded onto GST resin, SDS-PAGE was used to verify after 24 h digestion. The 3 C-digested proteins were eluted by GST buffer over 4 CV at 1 mL/min. The concentrated sample was then injected onto a Superdex 75 16/600 column (2 M NaCl, 1 x PBS, pH 7.5). The ubiquitin peak was eluted at an elution volume of 86.08 mL. Each fraction was analyzed by 15% SDS-PAGE. The peak fractions were pooled according to SDS-PAGE analysis and concentrated up to ∼ 10 mg/mL, aliquoted and flash-frozen in liquid nitrogen and stored in the refrigerator at -80^o^C for long-term storage.

### Expression and purification of mSWI/SNF complexes

Mammalian SWItch/Sucrose Non-Fermentable (mSWI/SNF) complexes, is a subfamily of ATP-dependent chromatin remodeling complexes. The mSWI/SNF complexes include the BRG1-associated factor (BAF), in its canonical (cBAF) and noncanonical (ncBAF) subtypes, were expressed and purified according to the previous work [34]. Briefly, the plasmids consisting of the BAF subunits were co-transfected to HEK Expi293F cells using PEI, then incubated at 37 °C for 72 h. The BAF subunits were sub-cloned into pcDNA34 vectors containing no-tag or N-terminal Strep-tag. Therefore, the cBAF and ncBAF complexes were eluted out by 3 mM α-desthiobiotin from the strep affinity column and further purified by ion exchange chromatography (Mono Q).

### Expression and purification of Dot1L

Wild-type Dot1L (1-420) was expressed and purified from the 8His-TEV-Dot1L plasmid according to the established work [35]. Dot1L plasmid was transformed into BL21(DE3) competent *E. coli* cells. Dot1L was expressed as soluble protein by induction with 0.5 mM IPTG at 18 °C overnight, and the protein was purified through Histrap column (GE). Eluted Dot1L protein was digested with TEV protease (NEB) overnight at 4^o^C to cleave off his-tag. After tag was cleaved off, sample was purified through HiTrap SP column (GE). Purified Dot1L protein was then concentrated, flash frozen in liquid nitrogen and stored in -80^o^C for future use.

### Cross-linking and purification of the Ubiquitinated histone octamers

The reaction of 1,3-dibromoacetone with ubiquitin G76C and histone octamer. All reagents were cooled to 4 °C before the reaction. In a typical reaction, H2AK119C histone octamer (100 µM, 14 mg) and 8 equivalents of ubiquitin (1 mM, 10 mg) were mixed and 10 equivalents of 1,3-dibromoacetione in DMF (500 mM, 0.58 µL) added and the solution incubated at 4 °C for 3 h. The reaction was quenched using 50 mM β-mercaptoethanol and analyzed by SDS-PAGE and Western blot. The other cross-linking of histone octamers and ubiquitin or ubiquitin-like protein (SUMO3, UFM1, and NEDD8) used the same procedure.

After cross-linking the reaction mixture was purified over the superdex 200 column to separate ubiquitin and histone octamers. The purified octamer products were further purified using HisTrap affinity chromatography. The bound proteins were eluted by increasing the imidazole concentration from 0 mM to 500 mM linearly over 30 CV at 2 mL/min. Each fraction was analyzed by SDS-PAGE and Western blot. The peak fractions were pooled according to SDS-PAGE analysis and concentrated up to 10 mg/mL, yielding ∼ 7.8 mg of ubiquitinated histone octamers with a 56% overall yield. Then the protein was aliquoted and flash-frozen in liquid nitrogen and stored in the refrigerator at -80^o^C for long-term storage. The purified H2AK119ub and H2BK120ub histone octamers were further characterized through tandem mass spectrometry (MS/MS) and size exclusion chromatography. And the molecular weight of ubiquitin monomer and di-ubiquitin after crosslinking were characterized through SEC-MALS experiment (Size Exclusion Chromatography coupled with Multi-Angle Light Scattering). The results can be found in Supplementary information Figure S7. Additionally, the H2AK119ub histone octamer from classical method [16] and fusion method [36] were also performed as control.

### Preparation of nucleosomes

Mono-nucleosome was reconstituted from ubiquitinated octamer and 147 bp DNA containing Widom 601 positioning sequence with optional flanking 38 bp DNA at either end (38N38). The DNA sequence for assembly of 38N38 nucleosome is (5-strand: AACAGCGACCTGAATTCTGGACCCTATACGCGGCCGCC*CTGGAGAATCCCGGTGCCGAGGCCGCTCAATTGGTCGTAGACAGCTCTAGCACCGCTTAAACGCACGTACGCGCTGTCCCCCGCGTTTTAACCGCCAAGGGGATTACTCCCTAGTCTCCAGGCACGTGTCAGATATATACATCCTGT*TCTAGAGCGGCCGCCACCGCGGTGGAGCTCCAGCTTTT, the ‘601’ positioning sequence is underlined). DNA fragments for assembly of mono-nucleosome were prepared by PCR and purified by ion exchange column. Peak fractions were collected for mono-nucleosome assembly according to the previous method [13, 15]. To calculate the nucleosome formation efficiency, we determine the concentration of nucleic acids before and after the reconstitution of nucleosomes. The formula used is as follows, with each experimental group consisting of three or four parallel sets.

$${\text{E}\text{f}\text{f}\text{i}\text{c}\text{i}\text{e}\text{n}\text{c}\text{y}}_{\left(\text{\%}\right)}=\frac{\text{N}\text{U}\text{C} \text{c}\text{o}\text{n}\text{c}\text{e}\text{n}\text{t}\text{r}\text{a}\text{t}\text{i}\text{o}\text{n}}{\text{I}\text{n}\text{t}\text{i}\text{a}\text{l} \text{c}\text{o}\text{n}\text{c}\text{e}\text{n}\text{t}\text{r}\text{a}\text{t}\text{i}\text{o}\text{n}}*100\%$$

### Nucleosome sliding assay

A nucleosome sliding assay was performed as previously described [34]. The BAF complexes (100 nM) were incubated with 38N38 nucleosome (100 nM) at 37 °C for 1 h in 20 µL reaction buffer containing 25 mM Tris-HCl, pH 8.0, 30 mM KCl, 5 mM MgCl_2_, 0.1 mg/mL BSA, 2% Glycerol and 3 mM ATP. The reactions were stopped by adding excess competitor plasmids (∼ 400 ng) and analyzed by native polyacrylamide gel electrophoresis (Native-PAGE). Gels were stained with GelRed and Coomassie blue and scanned on an automatic digital gel image analysis system.

### Restriction-enzyme accessibility assays (REAA)

Nucleosome restriction-enzyme accessibility assays were performed at 37 °C, as previously described [37]. 100 nM 38N38 H2AK119ub nucleosomes from NDHOU and classical methods were incubated with 50 nM ncBAF, 3 mM ATP and 100 U of HhaI in 20 µL reaction buffer (25 mM HEPES, pH 7.6, 30 mM KCl, 5 mM MgCl_2_, 2% Glycerol, and 0.1 mg/mL BSA). The reactions were taken at different time points and quenched with 2 × Stop buffer (50 mM HEPES, pH 7.6, 1.2% SDS, 40 mM EDTA, and 0.2 mg/mL proteinase K). Reaction mixtures were then incubated at 55 °C for 20 min to deproteinate the samples. The samples were running on 5% Native-PAGE in 0.2 × TBE for 60 min at 100 V on ice. Gels were stained with GelRed, scanned on an automatic digital gel image analysis system and quantified with the ImageJ. Original images of gels after inversion can be found in Supplementary information S14.

### Electromobility shift assays (EMSA)

The electromobility shift assays were performed for testing the application of the ubiquitinated nucleosomes. The Dot1L (1-420) and H2BK120 ubiquitinated nucleosomes were used to the assay. Briefly, 0.4 µM H2BK120 ubiquitinated nucleosomes was mixed with increasing amounts of Dot1L (0.4 µM, 0.8 µM, 1.6 µM, 3.2 µM, 6.4 µM, 12.8 µM) in EMSA buffer (20 mM HEPES, pH 7.5, 100 mM NaCl, 2% Glycerol, 100 mM DTT). The reaction mixtures were incubated at 4^o^C for 1 h to allow complex formation. Samples were then resolved on native 5% Native-PAGE electrophoresis in 0.2 x TAE buffer for 100 min at 100 V and stained with the PAGE GelRed nucleic acid gel stain and Coomassie blue stain.

### Histone methyltransferase assay (HMT)

The histone methyltransferase assay was performed for testing the application of the ubiquitinated nucleosomes. Wild type Dot1L (100, 200, or 400 nM) were incubated with H2BK120 ubiquitinated nucleosomes (400 nM), both unmodified and H2BK120-H3K79M ubiquitinated nucleosomes are used as controls in HMT assay buffer (20 mM Tris pH 8.0, 50 mM NaCl, 1 mM DTT, 1 mM MgCl_2_) in the presence of S-adenosyl methionine SAM (50 or 200 µM) at 30ºC for 1 h in 20 µL final volume. The reactions were stopped by SDS-loading buffer and the proteins were separated by 15% SDS-PAGE and transferred to 0.2-µm polyvinylidene difluoride PDVF membrane (Bio-Rad). The H3K79 methylation level was determined by incubating the membranes with anti-Histone H3 (mono + di + tri methyl K79) for 2 h at 25 °C. The Western blots were developed using ECL reagent and imaged in the ChemiDoc system (Bio-Rad).

## Results

### Design and optimization of conditions to conjugate ubiquitin into histone octamers

DCA/DBA-mediated cross-linking has been successfully employed to conjugate G76C ubiquitin to denatured H2A/H2B histones (e.g., H2AK119C or H2BK120C) through the sulfhydryl group of mutated cysteine residues [16, 26]. We hypothesized that a similar reaction could facilitate the incorporation of G76C ubiquitin into soluble histone octamers corresponding cysteine mutation. To apply the DCA/DBA-mediated conjugation under non-denaturing conditions, recombinant histone octamers and ubiquitin were expressed from *E. coli* and purified to homogeneity using a conventional two-step purification (Figure S1 and S2). These macromolecules were subsequently covalently linked using a DCA-mediated reaction, which was further quenched by 50 mM β-mercaptoethanol. The resulting product, indicative of the ubiquitylated histone derivative, was identified on SDS-PAGE by a band commensurate with its anticipated molecular weight (Figure S3).

The cross-linking reaction described above utilizes a bis-thio-acetone (BTA) linker to connect two cysteine residues. Notably, wild-type core nucleosome/octamer contains only a single conserved cysteine (H3C110) that is internally located within the nucleosome structure (Figure S4). We hypothesize that H3C110 is inaccessible to DCA-mediated reactions, obviating the need for this residue’s mutation. The internal positioning of cysteine in H3 histone prevents its ubiquitination, as confirmed by our experiments with DBA treatment (Figure S5). Therefore, it is feasible to introduce a cysteine mutation at any desired site on H2A/H2B tails (e.g., H2AK119C or H2BK120C) and, upon the treatment with DCA/DBA, the mutated cysteine can be linked with G76C of ubiquitin.

To enhance the NDHOU method’s efficiency and yield, we optimized various parameters, including molar ratio of reactants, pH, cross-linking reagent and concentration of DMF solvent (Fig. 2B and S6). DBA/DCA-mediated crosslinking proved to prefer a neutral pH compared to the alkaline conditions favored by halogenation reactions. Optimal conditions were established as 1: 8: 10 ratio of histone octamers to ubiquitin to crosslinking agent at pH 7.5, at 4 °C for three hours, and Western blot was used to identify the majority of H2A linked to ubiquitin after crosslinking under the optimal condition (Fig. 2C). Furthermore, DBA was preferred over DCA to minimize reaction rate reductions caused by chlorine in DCA and sodium chloride.

### Reconstitution of the Ubiquitinated Nucleosome in Nondenaturing conditions

**Fig. 3:**
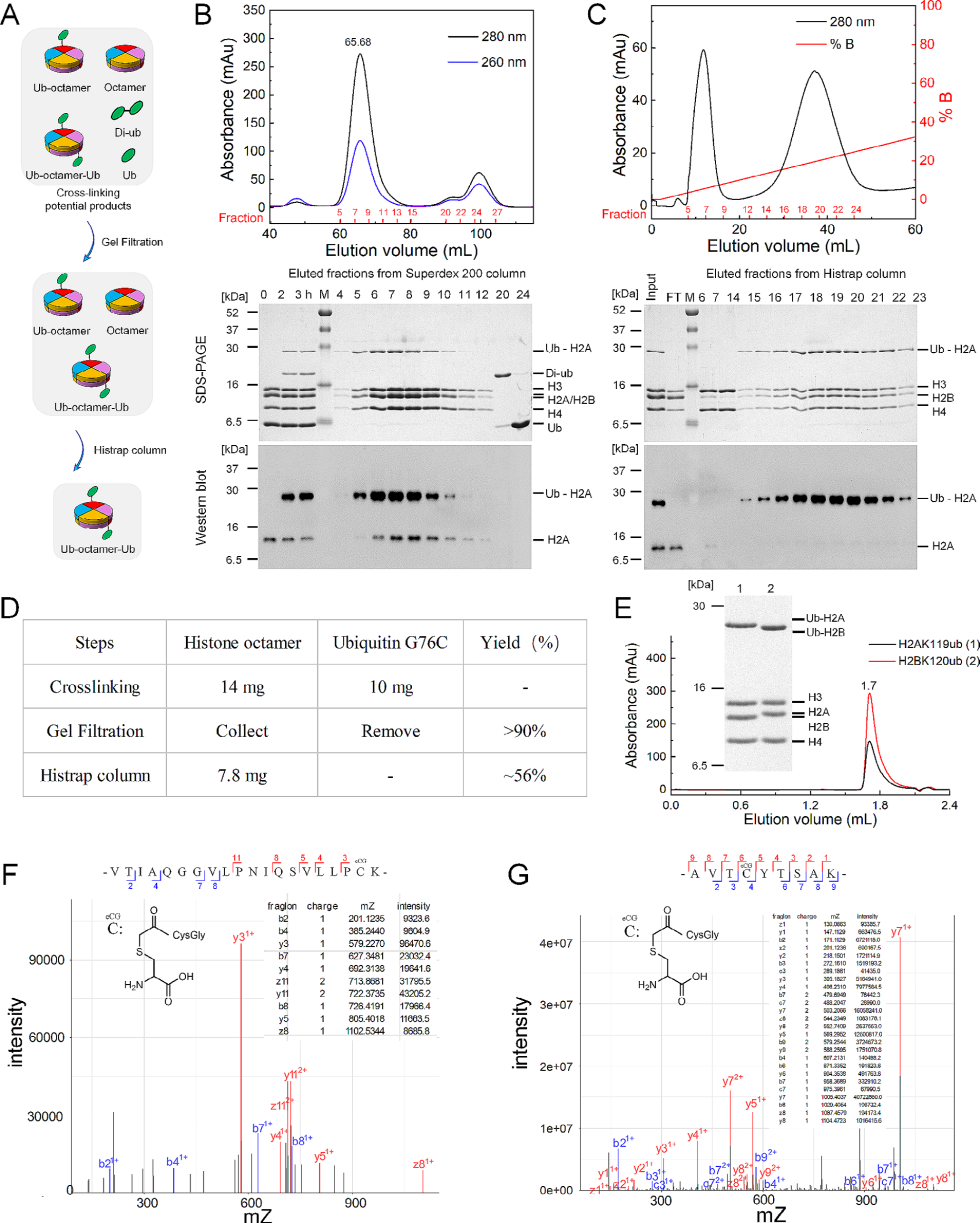
Purification and characterization of H2AK119 ubiquitinated histone octamer. **A** Schematic of the cross-linking reaction for purification of potential ubiquitination products; **B** Size exclusion chromatography demonstrating the separation of histone octamers and ubiquitin from the cross-linking reaction; **C** The gradient elution chromatography confirming the effective separation of ubiquitinated histone octamer from unmodified octamers; **D** The steps and yields for obtaining ubiquitinated histone octamers by NDHOU method; **E** Size-exclusion chromatography and SDS-PAGE analysis verifying the high purity of H2AK119 and H2BK120 ubiquitinated histone octamers; **F** MS/MS analysis of H2AK119ub and **G** H2BK120ub confirmed the installation of the ubiquitin at the correct position

We proceeded to explore whether the synthesized ubiquitinated histone octamer could successfully assemble into a nucleosome using practical methodologies. The purity and scalability of the synthesized ubiquitinated histone octamer are essential for efficient nucleosome reconstitution. To this end, size exclusion chromatography was applied to separated histone octamers from any free ubiquitin and di-ubiquitin (Fig. 3A and B). And the molecular weight of ubiquitin and di-ubiquitin were further characterized through SEC-MALS (Figure S7). It was also necessary to differentiate ubiquitinated octamers from a minority of unmodified ones. To achieve this, we engineered a 6x His-tag to the N-terminus of ubiquitin, enabling the selective removal of un-ubiquitinated octamers using a HisTrap affinity column (Fig. 3C). The ubiquitination of histone H2A was confirmed by Western blotting. Remarkably, the NDHOU method achieves an 56% overall yield after chemical synthesis and two-step purification, producing approximately 7.8 mg of ubiquitinated histone octamers (Fig. 3D). In contrast, prior studies typically report yields of less than 10% before the refolding step [38], with the highest yield for a single histone protein recorded at 55% [16]. In our conventional experiments, the efficiency of producing H2AK119ub histone reached about 44%, with an overall efficiency of 21% after reconstituting the ubiquitinated histone octamers (Figure S8). We further extended this approach to other histones, specifically H2BK120, resulting in the successful synthesis and isolation of H2BK120ub and H2BK120ub-H3K79M histone octamers. The purity and ubiquitination were verified by SDS-PAGE and Western blotting (Fig. 3E and Figure S9). Tandem mass spectrometry (MS/MS) analysis of H2AK119ub and H2BK120ub octamer samples confirmed the precise installation of ubiquitin at the designated sites (Fig. 3F and G).

**Fig. 4:**
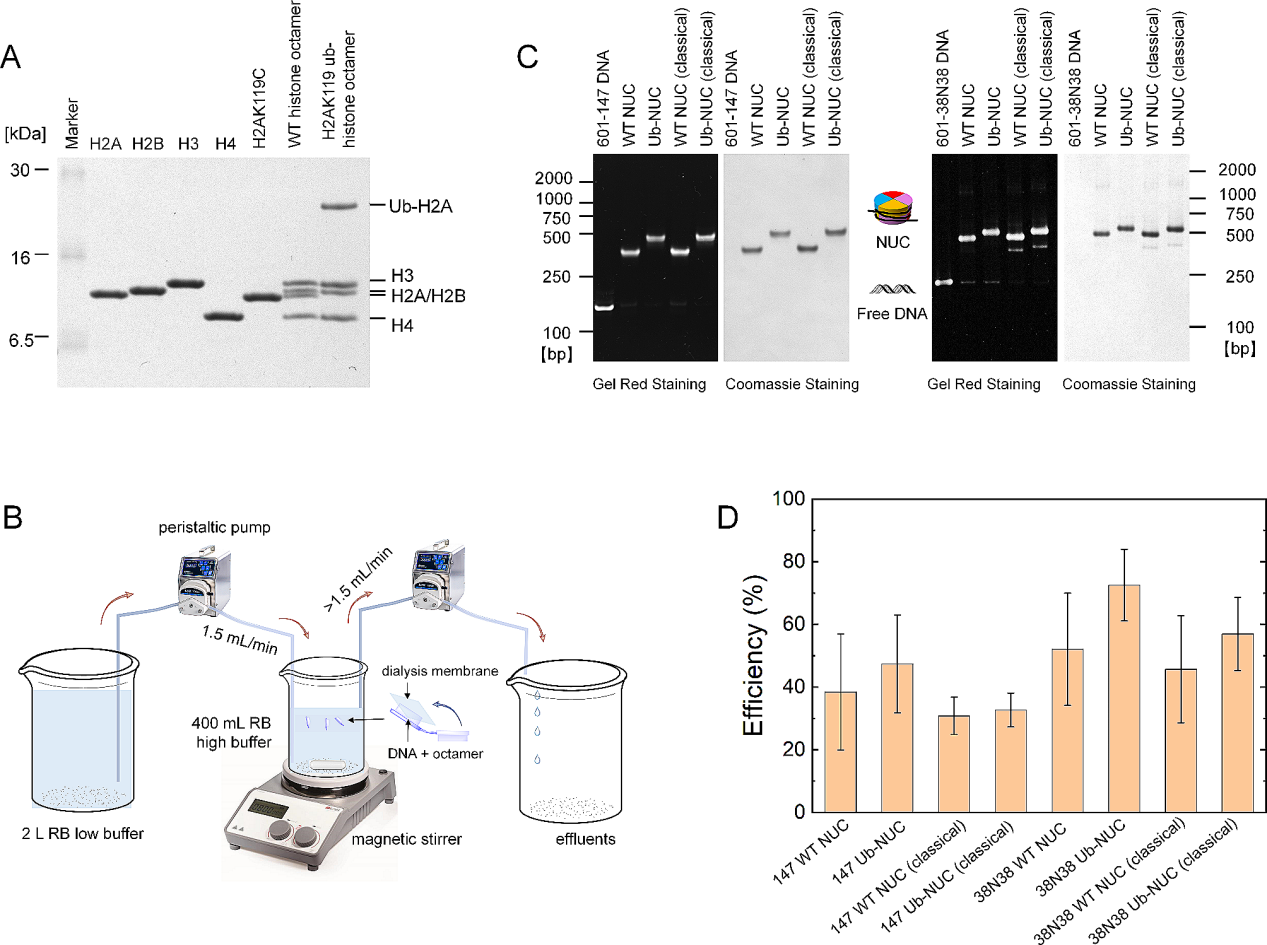
In vitro reconstitution of the ubiquitinated nucleosome under non-denaturing conditions. **A** SDS-PAGE analysis of the individual histones (H2A, H2B, H3, H4, and H2AK119C) purified from inclusion bodies and histone octamers (WT and H2AK119ub) obtained through refolding; **B** Diagram illustrating the experimental protocol for in vitro nucleosome reconstitution; **C** Native-PAGE analysis of various NCPs; WT NUC denotes unmodified nucleosomes, and Ub-NUC denotes ubiquitinated nucleosomes, with ‘classical’ referring to traditional reconstitution methods; **D** Comparison of nucleosome reconstitution efficiencies from different methodologies showing no significant differences, Error bars, s.d. (*n* = 4)

To ascertain whether nucleosomes prepared from ubiquitin-conjugated histone octamers exhibit characteristics akin to those of unmodified nucleosomes, we subjected both H2AK119-ubiquitinated and wild-type histone octamers to dialysis with either a 147 base pair (bp) W601 sequence [39, 40], or a version of W601 extended by additional 38 bp flanking DNA on both end (38N38) (Fig. 4A and B). The resulting mononucleosomes were examined by Native-PAGE to assess their electrophoretic properties and purity. As depicted in Fig. 4C, the band intensities on the PAGE indicate that the ubiquitinated nucleosomes reconstitute in quantities comparable to those of the unmodified nucleosomes. Additionally, ubiquitinated nucleosomes with extended flanking DNA exhibited band intensities similar to those without such extensions, indicating that the conjugated ubiquitin does not interfere with the flanking DNA.

We further compared nucleosomes reconstituted from both ubiquitinated and wild-type histones using the traditional denaturing method with those reconstituted *via* our rapid soluble approach. Analysis by the PAGE revealed that the nucleosomes reconstituted rapidly in solution displayed electrostatic characterizations similar to those reconstituted using the denaturing protocol (Fig. 4C). Additionally, we quantified the yield of recovered nucleosomes using different methods and evaluated the efficiency of these reconstitution approaches. While the efficiencies observed were not significantly different, the soluble reconstitution method proved to be marginally more effective than the denaturing and fusion method (Fig. 4D, Figure S10 and S11).

Taken together, our findings indicate that ubiquitinated octamers can be effectively assembled into nucleosomes with various DNA sequences, exhibiting properties comparable to those assembled using the denaturing approach. However, NDHOU approach offers the advantages of a more expedited reconstitution process and achieves marginally higher efficiency. In addition, nucleosomes containing H2BK120ub and the H2BK120ub-H3K79M variant were successfully reconstituted using NDHOU approach (Figure S12), underscoring its suitability for the rapid soluble reconstitution of a diverse array of nucleosome types.

### Functional analysis of Ubiquitinated nucleosomes obtained from nondenaturing conditions

**Fig. 5:**
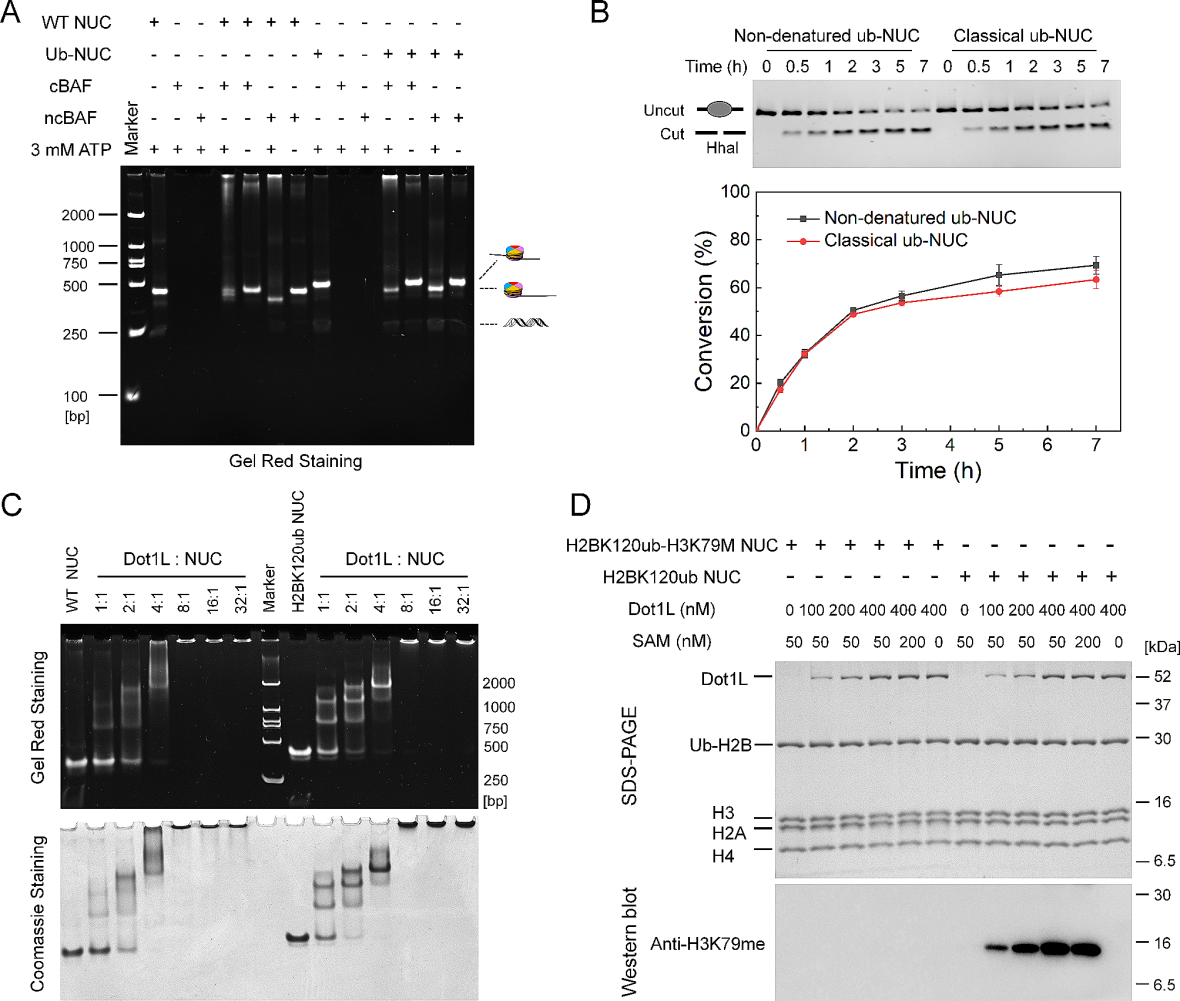
Functional characterization of ubiquitinated nucleosomes obtained under non-denaturing conditions. **A** Nucleosome sliding assays of H2AK119 ubiquitinated nucleosomes facilitated by cBAF and ncBAF. The Native-PAGE gel of the nucleosome sliding assays was stained by GelRed; **B** Representative gels of the restriction enzyme accessibility assays of non-denatured and classical ubiquitinated nucleosomes with ncBAF. The cut fractions were quantified from three independent assays in Figure S14. Data are mean and s.d. (*n* = 3 independent experiments); **C** The electromobility shift assay (EMSA) of nucleosomes (WT and H2BK120ub) with Dot1L. Increasing amounts of proteins (0.2, 0.4, 0.8, 1.6, 3.2, and 6.4 µM) were added to nucleosomes (0.2 µM). The results indicated increased binding affinity to H2BK120ub nucleosomes upon ubiquitin conjugation; **D** HMT assays with varying Dot1L (0, 100, 200, or 400 nM) or SAM (0, 50, or 200 nM) concentrations, in the presence of H2BK120ub-H3K79M or H2BK120ub nucleosomes. The results indicated that H2BK120 ubiquitinated nucleosomes from NDHOU approach undergo H3K79 methylation modification in the presence of Dot1L and SAM, with an enhanced H3K79 methylation observed as the concentration of Dot1L increases

To confirm the suitability of ubiquitinated nucleosomes synthesized *via* NDHOU approach for biochemical studies, we employed them as substrates in a chromatin remodeling assay. BAF complexes, which are part of the human SWI/SNF remodeling family, reposition nucleosomes along DNA in an ATP-dependent mechanism [34]. We produced two variants of the BAF complex (cBAF and ncBAF) and conducted the remodeling assays with both wild-type and ubiquitinated nucleosomes. Upon ATP addition, both BAF complexes successfully shifted nucleosomes from a central to an end position on the DNA, demonstrating the functionality of the ubiquitinated nucleosomes reconstituted by our NDHOU approach (Fig. 5A). The comparable remodeling of both unmodified and ubiquitinated nucleosomes indicates that the chemical reaction employed to generate ubiquitinated histone octamers does not have adverse effects on the soluble histone octamer, consistent with the clean installation of ubiquitin observed by mass spectrometry. To verify that non-denatured nucleosomes function comparably to conventional ones, we conducted restriction-enzyme accessibility assays to evaluate and measure the nucleosome sliding efficiencies between non-denatured and classical ubiquitinated nucleosomes. The comparable performance of 38N38 non-denatured and classical ubiquitinated nucleosomes under the ncBAF complex suggests that nucleosomes synthesized using the NDHOU method are effective substitutes for classical ubiquitinated nucleosomes in biochemical studies (Fig. 5B and Figure S13).

In addition to the stability and intact properties of our ubiquitinated nucleosomes, which are conducive to chromatin remodeling studies, it has been suggested that ubiquitin modifications of histone tails might be recognized and play a regulatory role. We generated nucleosomes with site-specific ubiquitination at H2BK120 and investigated whether these H2BK120ub nucleosomes are preferentially recognized by Dot1L, a histone H3 Lys79 methyltransferase whose activity is stimulated by histone H2B Lys120 ubiquitination [35, 41–43]. The electromobility shift assay (EMSA) assay was performed to analyse the affinity between Dot1L (1-420) and H2BK120ub nucleosome, or unmodified nucleosome. The results revealed that the attached ubiquitin markedly enhanced the affinity between Dot1L and the H2BK120ub nucleosome, suggesting that the installed ubiquitin is properly recognized by chromatin regulatory enzymes (Fig. 5C).

Although the ubiquitin installed by our rapid soluble approach demonstrates behaviour similar to its unmodified counterparts in binding assays, the sensitivity of chemical catalysis to small changes in the structure suggests that the ability of our ubiquitinated nucleosome to function in enzymatic assays presents a more rigorous validation of similarity. To investigate such cross-talk between ubiquitin modification and enzymatic functions (e.g., methyl transferase activity in Dot1L) in our synthesised nucleosomes, we performed histone methyltransferase (HMT) assays to measure H3K79 methylation levels (mono-, di-, and tri-methylation) in the presence of Dot1L and S-adenosyl methionine (SAM), using the H2BK120ub nucleosome synthesized by our approach as a substrate. As the negative control, the H3K79 was mutated to methionine to generate a H2BK120ub-H3K79M nucleosomes that are resistant to H3K79 methylation (Fig. 5D). In this assay, increasing concentrations of Dot1L led to efficient methylation of H3K79 on H2BK120ub nucleosomes, but with increased efficiency significantly when compared to unmodified nucleosomes, consistent with previous observations of unmodified and H2BK120ub nucleosomes produced by conventional denaturing approach [35, 44] (Figure S14). Therefore, we conclude that the ubiquitin incorporated into nucleosomes by our rapid soluble approach behaves in a functionally analogous manner to conventional ubiquitinated nucleosomes, even within the geometrically and chemically complex context of epigenetic regulatory events.

### Extending the application to ubiquitin-like modifications on all Core histones

**Fig. 6:**
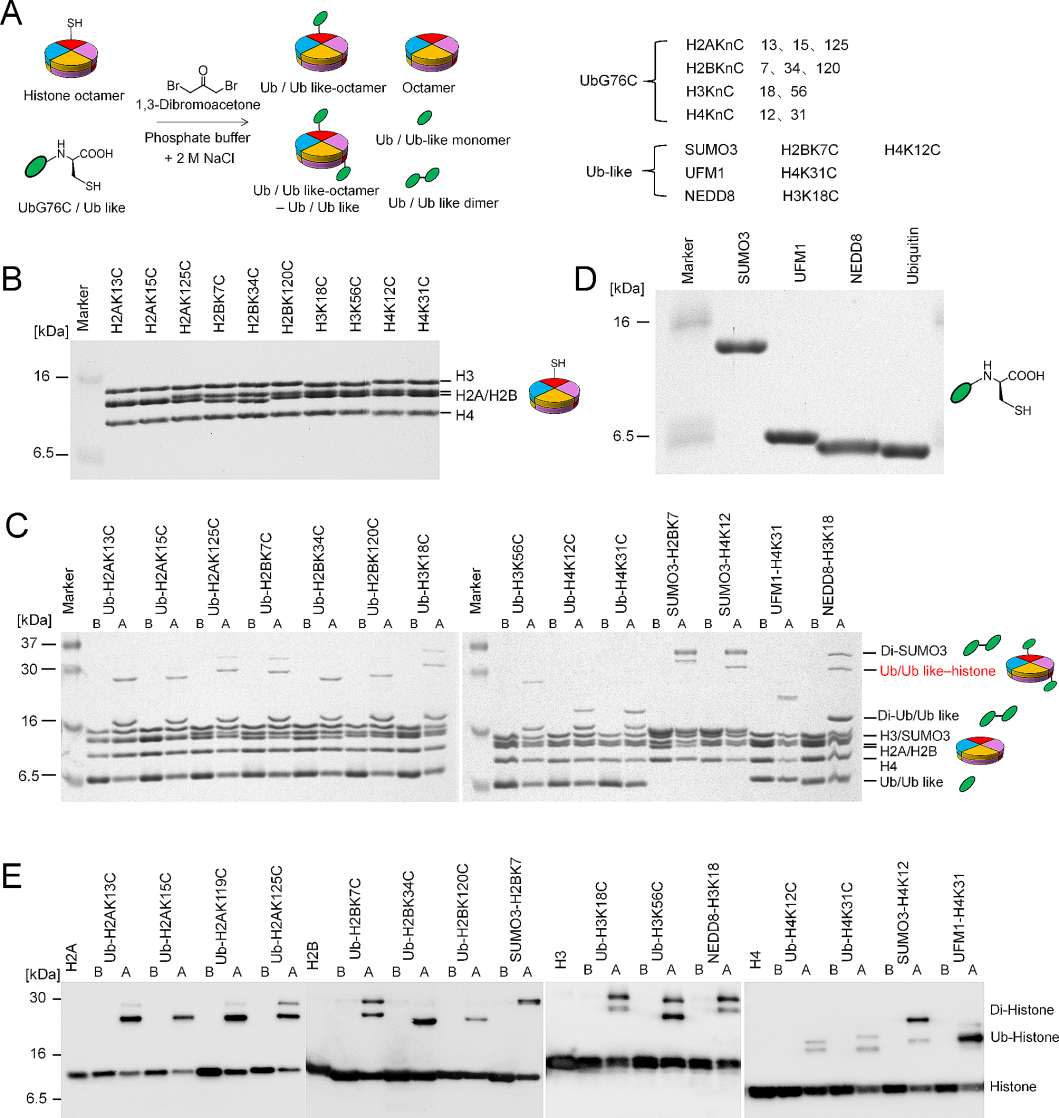
Extending the application to ubiquitin-like modifications on all core histones. **A** Schematic representation of DBA-mediated cross-linking method for introducing ubiquitin and ubiquitin-like modifications onto histone octamers in non-denaturing conditions; **B** SDS-PAGE analysis of the various histone octamers; **C** SDS-PAGE analysis of cross-linking reactions between histone octamers and ubiquitin or ubiquitin-like proteins; **D** SDS-PAGE analysis of ubiquitin and three ubiquitin-like proteins (SUMO3, UFM1, NEDD8); **E** Western blotting analysis of cross-linking reactions between histone octamers and ubiquitin or ubiquitin-like proteins, with ‘B’ indicating before and ‘A’ denoting after cross-linking

We expanded our methodology to include typical ubiquitylation sites across all four core histones by engineering a series of site-specific single cysteine mutants (Fig. 6A). These mutated octamers were then successfully purified and conjugated with ubiquitin under non-denaturing conditions (Fig. 6B and C and Figure S15), indicating this approach is versatile and can be applied to introduce ubiquitin modifications across all core histone tails. Ubiquitin-like modifiers, such as SUMO3, UFM1, and NEDD8, are known to be recruited to histones in response to DNA damage and other cellular pathways [5]. We aimed to determine whether NDHOU approach could be adapted for attaching ubiquitin-like modifications to histone octamers in non-denaturing conditions. Proteins including SUMO3, UFM1, and NEDD8 were initially overexpressed, purified (Fig. 6D), and then cross-linked to soluble histone octamers under the established non-denaturing conditions. Subsequent SDS-PAGE and Western blot analyses confirmed that the histone octamers were successfully modified with the ubiquitin-like proteins (Fig. 6C and E and Figures S16-S18). Notably, UFM1 and NEDD8 modifications were conjugated to H4 at lysine 31 and to H3 at lysine 18, respectively, which demonstrated these ubiquitin-like modifications can be successfully attached to all core histones.

Taken together, our results demonstrate that the NDHOU approach under non-denaturing conditions is not limited to ubiquitination of H2A/H2B but is also expected to apply to ubiquitin-like modifications on all four core histones. The latter suggests that this approach could potentially be extended to a broader range of modifications, such as methyl-lysine analogs [45], targeting specific sites on core histone tails (see Discussion).

## Discussion

Using co-expressed histones [18] and DBA-mediated chemical crosslinking strategies [16, 26], we have established a non-denaturing approach for the ubiquitylation of histone octamers, enabling direct incorporation of ubiquitin and ubiquitin-like modifications. We have demonstrated that mono-nucleosomes can be successfully reconstituted on these modified octamers. Functional analyses, including chromatin remodeling assays, restriction-enzyme accessibility assays (REAA), electromobility shift assays (EMSA), and histone methyltransferase (HMT) assays, have confirmed their effectiveness as substrates for nucleosome assembly and subsequent in vitro biochemical studies. In addition, we have effectively broadened this method to introduce ubiquitin modifications across all four core histones and have adapted it to accommodate three distinct ubiquitin-like modifications. These findings indicate that our approach holds considerable promise for research into epigenetic regulation.

Histone ubiquitination modification is emerging as an essential epigenetic regulator implicated in a range of biological processes. Procuring ample quantities of homogeneous ubiquitinated nucleosome substrates in vitro is pivotal for elucidating the role of histone ubiquitination in chromatin regulatory activities. Recently, high-efficiency chemical biology approaches have shown promise for introducing ubiquitin modifications into recombinantly reconstituted nucleosomes for in vitro biochemical studies [35, 46–48]. These approaches include total chemical synthesis utilizing native chemical ligation (NCL), semisynthesis leveraging expressed protein ligation (EPL), and cross-linking strategies employing recombinant proteins [19, 49–54]. Nevertheless, the complexity of chemical synthesis, particularly total synthesis and semisynthesis, involves techniques that are not commonly used in traditional biological laboratories, posing a barrier to broader research adoption [55].

A significant challenge in producing ubiquitylated nucleosomes for biophysical studies is the medium to large-scale synthesis of recombinant octamers. While the traditional method for synthesizing ubiquitinated histone octamers is effective, it is also labor-intensive and time-consuming [13, 15]. This method requires the individual expression and purification of the four core histones from inclusion bodies under denaturing conditions, followed by their refolding into histone octamers in high-salt buffers. Mueller-Planitz et al. have refined this approach, offering a more rapid purification process, yet the procedure still necessitates refolding dialysis steps [56]. A substantially more streamlined method has since been devised, allowing to produce recombinant histone octamers under non-denaturing conditions within a single day [18]. This innovative method utilizes a single polycistronic vector for the co-expression of all four histones in *E. coli*, facilitating the direct purification of the histone octamer. NDHOU approach aligns with prior strategies in producing a covalent bond akin to the native isopeptide linkage, with the DBA crosslinker proving more effective for ubiquitination than DCA [16, 26]. Additionally, our approach uniquely facilitates the modification of histone octamers under non-denaturing conditions, a departure from the predominantly denaturing conditions of existing methods [19, 20]. However, it is unfortunately limitation of our NDHOU method to add multiple ubiquitin to the octamer at same or different histones in one time due to the complexity of the products after cross-linking, which poses challenges during the purification stage.

Furthermore, this method could offer novel insights into the study of additional modifications, such as methylation and acetylation, in non-denaturing environments and may be adapted for modifying other folded proteins. Histone ubiquitination and ubiquitin-like modification, such as SUMOylation, are bulky post-translation modifications. Although ubiquitination is one the most abundant types of histone PTMs it’s crucial to note that mimicking this modification demands the addition of a ubiquitin group to the designated lysine residue, while other PTMs such as methylation, phosphorylation, and acetylation can be represented by amino acid mutations such as methionine, aspartic or glutamic acid and glutamine respectively [57, 58]. Several structural and functional studies have utilized this approach to answer significant biological questions [41, 59]. In disease conditions such as cancer, these histone mutations signify the constitutively modified residue regardless of any aberrations in the enzyme machinery responsible for carrying out or removing these modifications [60, 61]. Therefore, there is hope that these modifications including methylation, phosphorylation, and acetylation can be extensively utilized in our system. However, a constitutive ubiquitinated modification necessitates ubiquitin being covalently attached to the specific histone residue. To date, there’s no literature evidence supporting the notion that an amino acid mutation can represent the ubiquitinated state. Typically, the presence or the absence of histone ubiquitination highlights irregularities and anomalies in either the catalytic enzyme itself or the upstream regulatory machinery. Our study presents an alternative way to covalently attach the ubiquitin moiety onto the histone without any denaturing step; instead, we use the histone octamer in its native state.

PTMs associated with histones predominantly occur in the flexible and exposed histone tails. However, some core histone modifications have been extensively studied, including H3K79 methylation. One of the ways to mimic methylation is to install methyl lysine analog (MLA) [45]. The published study is well received and the approach has been utilized by many studies. However, the installation of MLA would require the denaturation of histone octamer. In 2019, Worden EJ et al., published a study based on the crosstalk of H2B ubiquitination and H3K79 methylation [41]. Our approach can be utilized to combine H2BK120 ubiquitination and H3K79MLA in histone octamer. We propose that the histones containing H3K79C and H2BK120C can be purified as an octamer, ubiquitination reaction using our method can be carried out on the native octamer, the ubiquitinated octamer can later be denatured to carry out the MLA reaction. Our method is favorable but is unfortunately limited to PTM combinations of core histone residues with bulky histone tail PTMs in the histone octamer.

## Conclusions

In conclusion, we have refined a protocol for the rapid production of ubiquitylated and ubiquitin-like modified (SUMOylation, NEDDylation, and UFMylation) histone octamers and their subsequent reconstitution into nucleosomes for in vitro studies. Notably, this process is conducted under non-denaturing conditions without the need for purifying individual histones, offering a less labor-intensive and higher-yielding alternative to established methods. The method capitalizes on the increased efficacy of the cross-linker DBA for coupling the two engineered cysteine residues on the co-expressed histone octamers with recombinant ubiquitin or ubiquitin-like proteins (SUMO3, NEDD8, and UFM1). Our observations indicate a marked enhancement in yield compared to traditional techniques, which can be largely ascribed to the accelerated chemical reaction rates. Optimization revealed that using a surplus of ubiquitin and cross-linker contributes positively to the modification efficiency of the histone octamers. A notable advantage of our approach is that it is conducted at physiological pH, conducive to protein stability. The ubiquitinated H2AK119 histone octamer was successfully integrated into nucleosomes without necessitating additional purification. Its biological functionality was substantiated through nucleosome sliding, REAA, EMSA, and HMT assays, indicating that the streamlined synthetic ubiquitinated nucleosomes are biologically active. These findings confirm that our DBA-facilitated synthetic approach for ubiquitinated and ubiquitin-like nucleosomes holds significant promise for producing substrates critical for epigenetic research.

### Electronic supplementary material

Below is the link to the electronic supplementary material.

Supplementary Material 1

## Data Availability

The original data presented in this study are included in the article/additional files, and further inquiries can be directed to the corresponding authors.
