# Supplementary material for: Rapid reconstitution of ubiquitinated nucleosome using a non-denatured histone octamer ubiquitylation approach
Source: Cell Biosci. 2024 Jun 17;14:81. doi: 10.1186/s13578-024-01265-x (PMC11184750; doi:10.1186/s13578-024-01265-x)
Supplement: Supplementary file 1 — Supplementary Material 1 [file 13578_2024_1265_MOESM1_ESM.docx]

**ADDITIONAL MATERIAL**

**Rapid Reconstitution of Ubiquitinated Nucleosome using a Non-Denatured Histone Octamer Ubiquitylation approach**

Weijie Li^1,†^, Peirong Cao^5,8,†^, Pengqi Xu^1^, Fahui Sun^2^, Chi Wang^2,4^, Jiale Zhang^2,6^, Shuqi Dong^2,6^, Jon R. Wilson^7^, Difei Xu^1^, Hengxin Fan^1^, Zhenhuan Feng^2,6^, Xiaofei Zhang^2,3^, Qingjun Zhu^2^, Yingzhi Fan^2^, Nick Brown^7^, Neil Justin^7^, Steven J Gamblin^7^, He Li^3^, Ying Zhang^1*^, and Jun He^2,3*^

1 Tomas Lindahl Nobel Laureate Laboratory, The Seventh Affiliated Hospital, Sun Yat-Sen University, Shenzhen, 518107, China.

2 CAS Key Laboratory of Regenerative Biology, Guangdong Provincial Key Laboratory of Stem Cell and Regenerative Medicine, GIBH-HKU Guangdong-Hong Kong Stem Cell and Regenerative Medicine Research Centre, GIBH-CUHK Joint Research Laboratory on Stem Cell and Regenerative Medicine, Guangzhou Institutes of Biomedicine and Health, Chinese Academy of Sciences, Guangzhou 510530, China

3 Key Laboratory of Biological Targeting Diagnosis, Therapy and Rehabilitation of Guangdong Higher Education Institutes, The Fifth Affiliated Hospital of Guangzhou Medical University, Guangzhou 510799, China

4 School of Life Sciences, University of Science and Technology of China, Hefei 230026, China

5 Department of Neuroscience, City University of Hong Kong, Kowloon Tong, Hong Kong SAR, P.R. China

6 University of Chinese Academy of Sciences, Beijing 100049, China

7 Francis Crick Institute, 1 Midland Road, London, NW1 1AT, United Kingdom.

8 School of Chinese Medicine, Hong Kong Baptist University, Hong Kong, China

†Weijie Li and Peirong Cao have contributed equally to this work.

*Correspondence: he_jun@gibh.ac.cn (J.H.), Zhangy856@mail.sysu.edu.cn (Y.Z.).

SUPPLEMENTAL METHODS

Materials

Isopropanol, dimethylformamide (DMF), dimethyl sulfoxide (DMSO) and all other organic solvents used in this study were available from the Guangzhou Chemical Reagent Company. 1,3-dichloroacetone (DCA), 1,3-dibromoacetone (DBA), Isopropyl β-D-Thiogalactoside (IPTG), α-desthiobiotin, S-(5-Adenosyl)-L-methionine (SAM) and all other chemical reagent were available from the Shanghai Aladdin Biochemical Technology Company. Anti-Histone H2A (Cat# AP13062a), Anti-Histone H2B (Cat# AP19790b), Anti-Histone H3 (mono+di+tri methyl K79) (Cat# CY5483-50) were available from Abcepta and Abways. GST resin, heparin column, superdex 200 16/600 column, HisTrap column, strep column, and Mono Q, HiTrap SP HP and all other reins were bought from Cytiva (formerly GE Life Sciences). *E Coli* BL21 (DE3), Rosetta (DE3), and all other competent cells were purchased from Shanghai AngYu Biotechnologies company. Water used in this work was obtained from a Millipore Milli-Q water purification system with an electric resistance of 18.4 MΩ cm.

**Method details**

**Construction of protein expression plasmid**

The core histones (H2A, H2B, H3, and H4) from *Xenopus Laevis* were cloned in series into the pRSFDuet with the H2B, H3 and H4 coding sequence preceded by a ribosome binding site. Point mutations to introduce cysteine residues for cross-linking were introduced by PCR.

**H2AK119C histone octamer:** H2AK119C point mutation was introduced to *Xenopus Laevis* H2A gene cloned in pRSFDuet plasmid vector encoding all four core histones (H2A, H2B, H3, and H4) by PCR. Other histone octamers were obtained using the same method.

H2AK119C Amino acid sequence:

**(H2A)**MGMSGRGKQGGKTRAKAKTRSSRAGLQFPVGRVHRLLRKGNYAERVGAGAPVYLAAVLEYLTAEILELAGNAARDNKKTRIIPRHLQLAVRNDEELNKLLGRVTIAQGGVLPNIQSVLLPCKTESSKSAKSK***(H2B)**MAKSAPAPKKGSKKAVTKTQKKDGKKRRKTRKESYAIYVYKVLKQVHPDTGISSKAMSIMNSFVNDVFERIAGEASRLAHYNKRSTITSREIQTAVRLLLPGELAKHAVSEGTKAVTKYTSAK***(H3)**MARTKQTARKSTGGKAPRKQLATKAARKSAPATGGVKKPHRYRPGTVALREIRRYQKSTELLIRKLPFQRLVREIAQDFKTDLRFQSSAVMALQEASEAYLVALFEDTNLCAIHAKRVTIMPKDIQLARRIRGERA***(H4)**MSGRGKGGKGLGKGGAKRHRKVLRDNIQGITKPAIRRLARRGGVKRISGLIYEETRGVLKVFLENVIRDAVTYTEHAKRKTVTAMDVVYALKRQGRTLYGFGG*

**Ubiquitination (G76C):** An N-terminal His tag was engineered into ubiquitin, SUMO3, NEDD8 and UFM1 and these constructs were cloned into the pGEX-6P1 plasmid. C-terminal cysteine residues introduced by PCR.

G76C point mutation was introduced to Ubiquitin gene cloned in pGEX-6P-1 plasmid vector. Amino acid sequence as follows:

MQIFVKTLTGKTITLEVEPSDTIENVKAKIQDKEGIPPDQQRLIFAGKQLEDGRTLSDYNIQKESTLHLVLRLRGC

**SUMO3 (G92C):** G92C point mutation was introduced to SUMO3 gene cloned in pGEX-6P-1 plasmid vector. Amino acid sequence as follows:

MSEEKPKEGVKTENDHINLKVAGQDGSVVQFKIKRHTPLSKLMKAYCERQGLSMRQIRFRFDGQPINETDTPAQLEMEDEDTIDVFQQQTGC

**UFM1 (G83C):** G83C point mutation was introduced to UFM1 gene cloned in pGEX-6P-1 plasmid vector. Amino acid sequence as follows:

MSKVSFKITLTSDPRLPYKVLSVPESTPFTAVLKFAAEEFKVPAATSAIITNDGIGINPAQTAGNVFLKHGSELRIIPRDRVC

**NEDD8 (G76C):** G76C point mutation was introduced to NEDD8 gene cloned in pGEX-6P-1 plasmid vector. Amino acid sequence as follows:

MLIKVKTLTGKEIEIDIEPTDKVERIKERVEEKEGIPPQQQRLIYSGKQMNDEKTAADYKILGGSVLHLVLALRGC

**QUANTIFICATION AND STATISTICAL ANALYSIS**

Origin 2018, ChemBioDraw Ultra 14.0, Visio 2021, ImageJ, and Chimera-1.16 was used to analyse and process data.

**Additional Material**


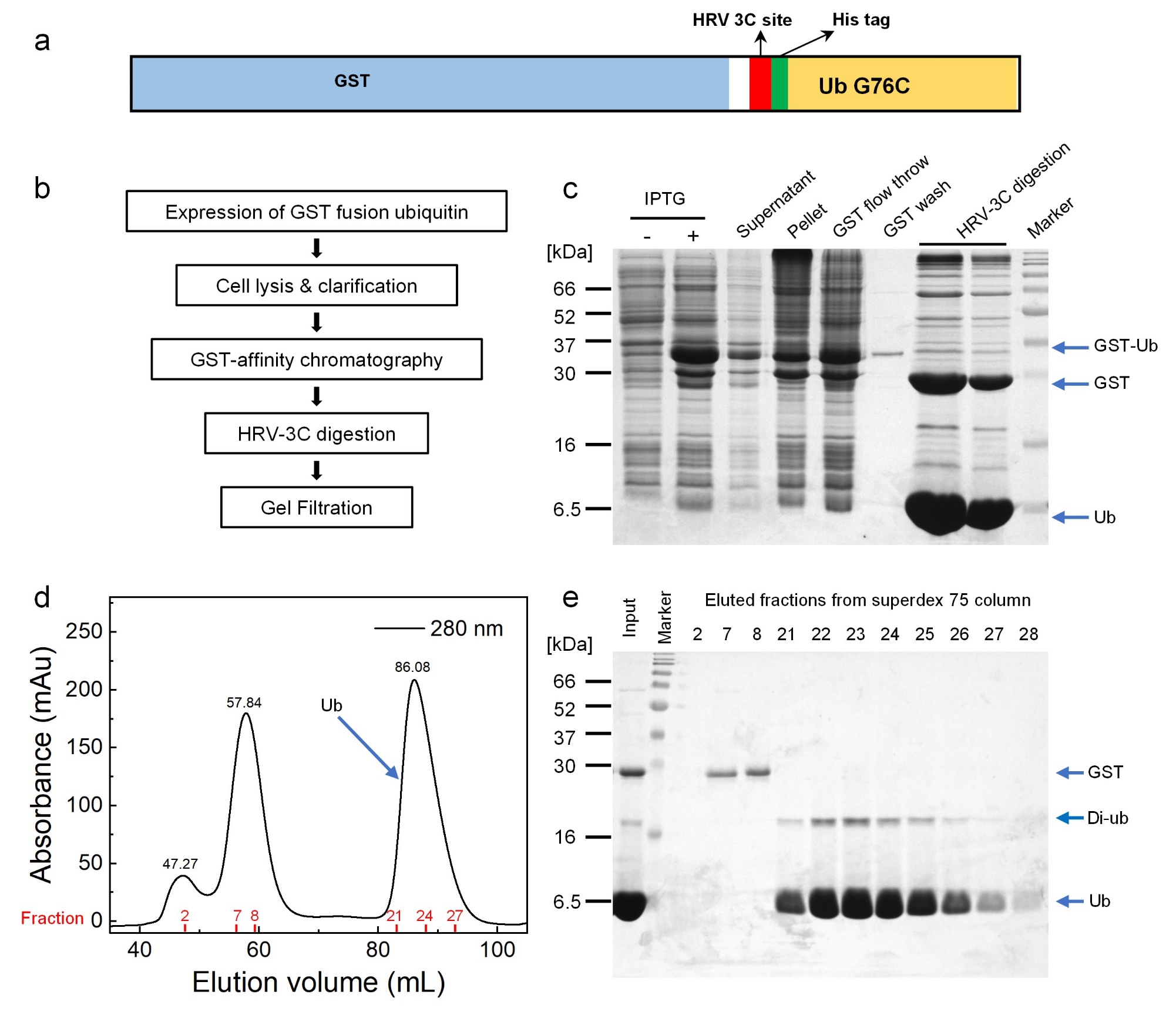


Figure S1. The expression and purification of ubiquitin (G76C). (a) Schematic of the GST fusion ubiquitin (G76C) expression vector. (b) The overall procedure of ubiquitin expression and purification. (c) – and + represent before and after induced expression by IPTG, SDS-PAGE gel shows the purification process of ubiquitin through the GST affinity chromatography and after HRV-3C digestion. (d) Ubiquitin was finally purified over a Superdex 75 column. (e) SDS–PAGE gel shows that stoichiometric ubiquitin was eluted at 86.08 mL.


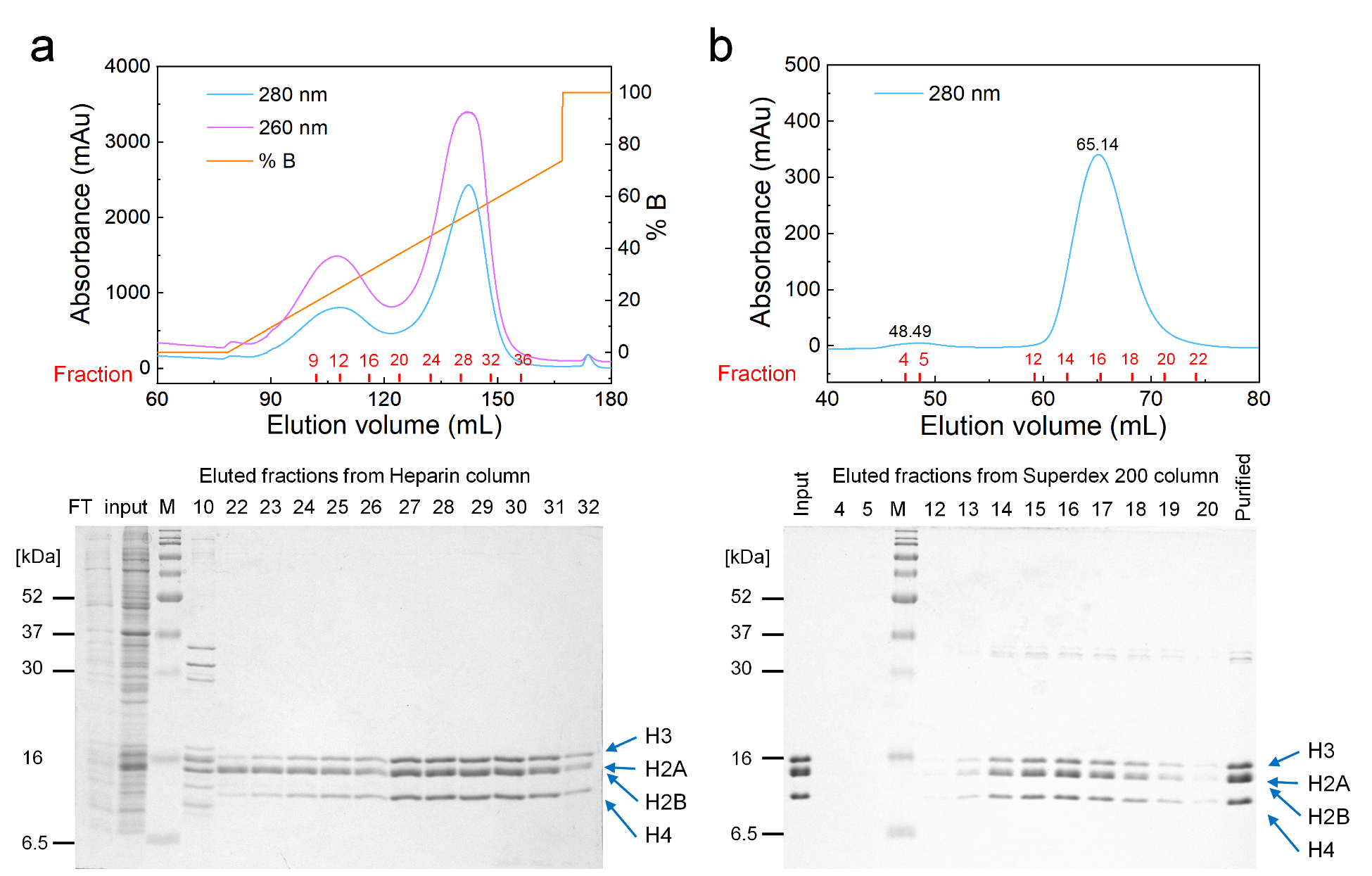


Figure S2. The purification of H2AK119C histone octamer. (a) Histone octamer (H2AK119C) was purified over a Heparin column, SDS-PAGE gel shows that octamer elutes at 56% B, (b) The histone octamer eluted from heparin column at peak2 was further purified by a Superdex 200 column, the gel filtration and SDS-PAGE gel show that stoichiometric histone octamer elutes at 65.14 mL.


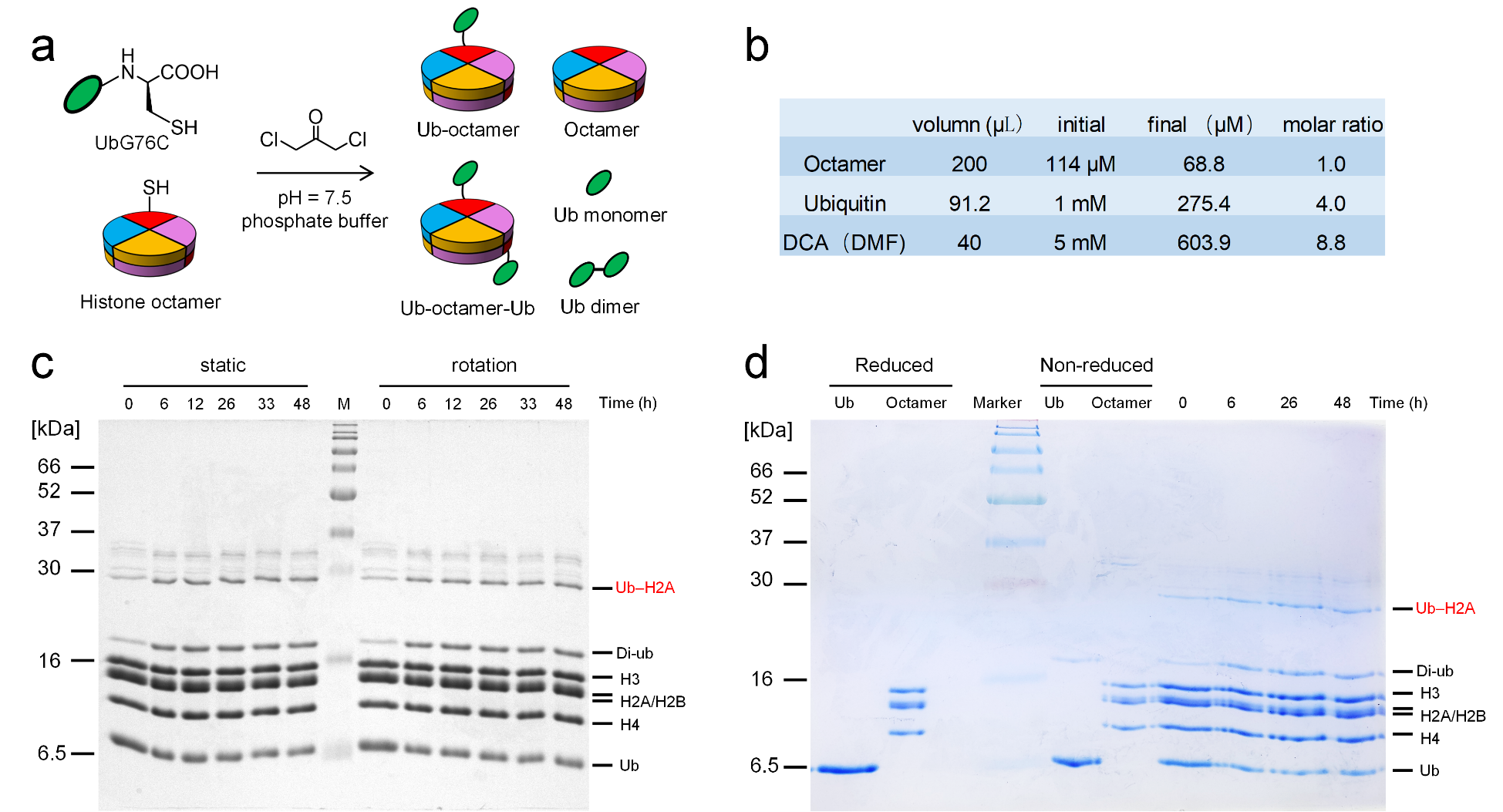


Figure S3. Supplementary materials for Fig. 2. (a) Schematic of DCA-assisted crosslinking of ubiquitin (G76C) and histone octamer (H2AK119C). (b) The initial and final concentrations of histone octamer (H2AK119C), ubiquitin (G76C), and 1,3-dichloroacetone (DCA) and their molar ratio. (c) SDS-PAGE analysis of the histone octamer, ubiquitin and DCA after incubation on an ice bath at the different time points (0, 6, 12, 26, 33, and 48 h) under static and rotation conditions. (d) SDS-PAGE analysis of the histone octamer and ubiquitin before (reduced and non-reduced) and after incubation on an ice bath at the different time points (0, 6, 26, and 48 h) under static conditions.


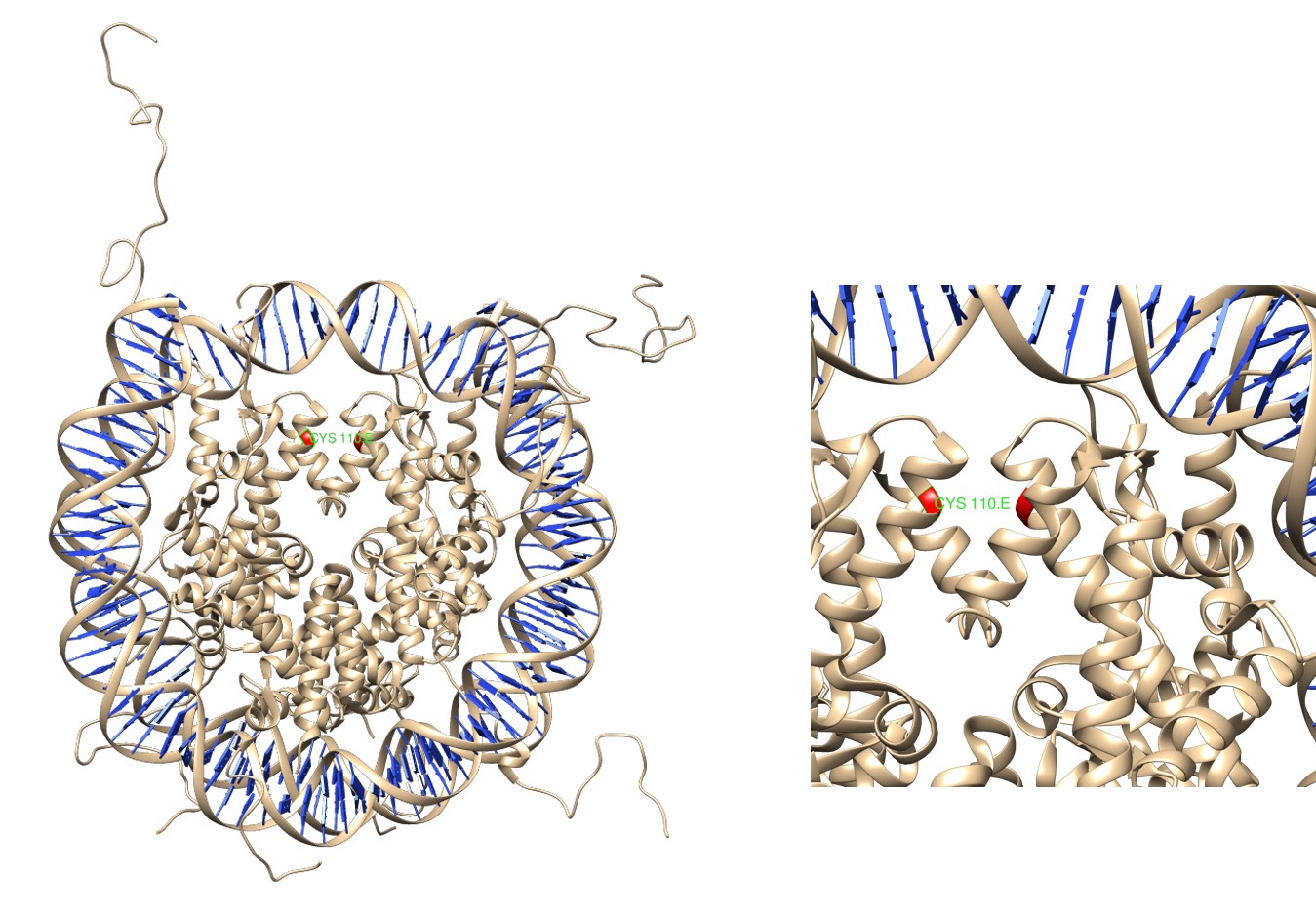


Figure S4. Supplementary materials for Fig. 2. The location of H3C110 was marked by red in 1.9 Å resolution crystal structure of nucleosome. (PDB:1KX5)


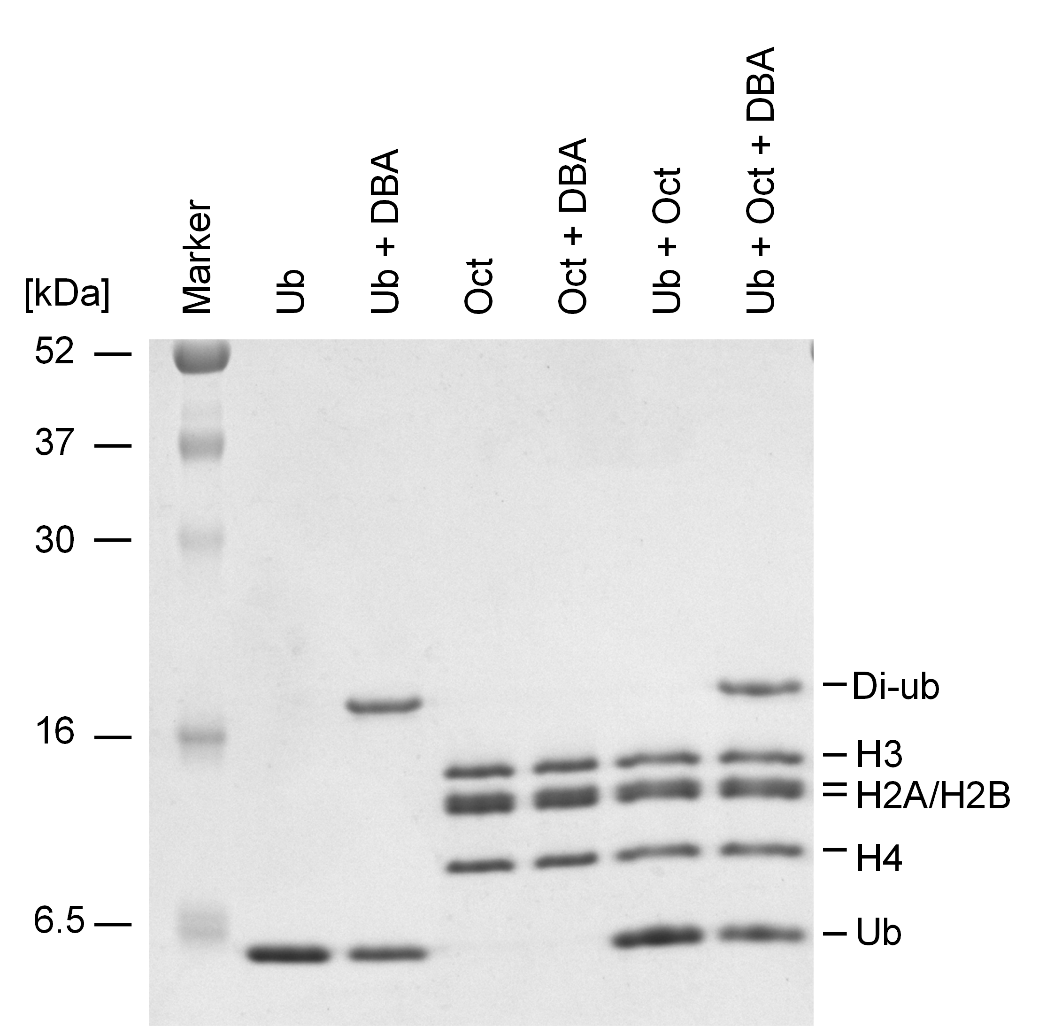


Figure S5. Supplementary materials for Fig. 2. DBA-assisted crosslinking of ubiquitin (G76C), WT histone octamer, as well as ubiquitin and histone octamer, respectively.


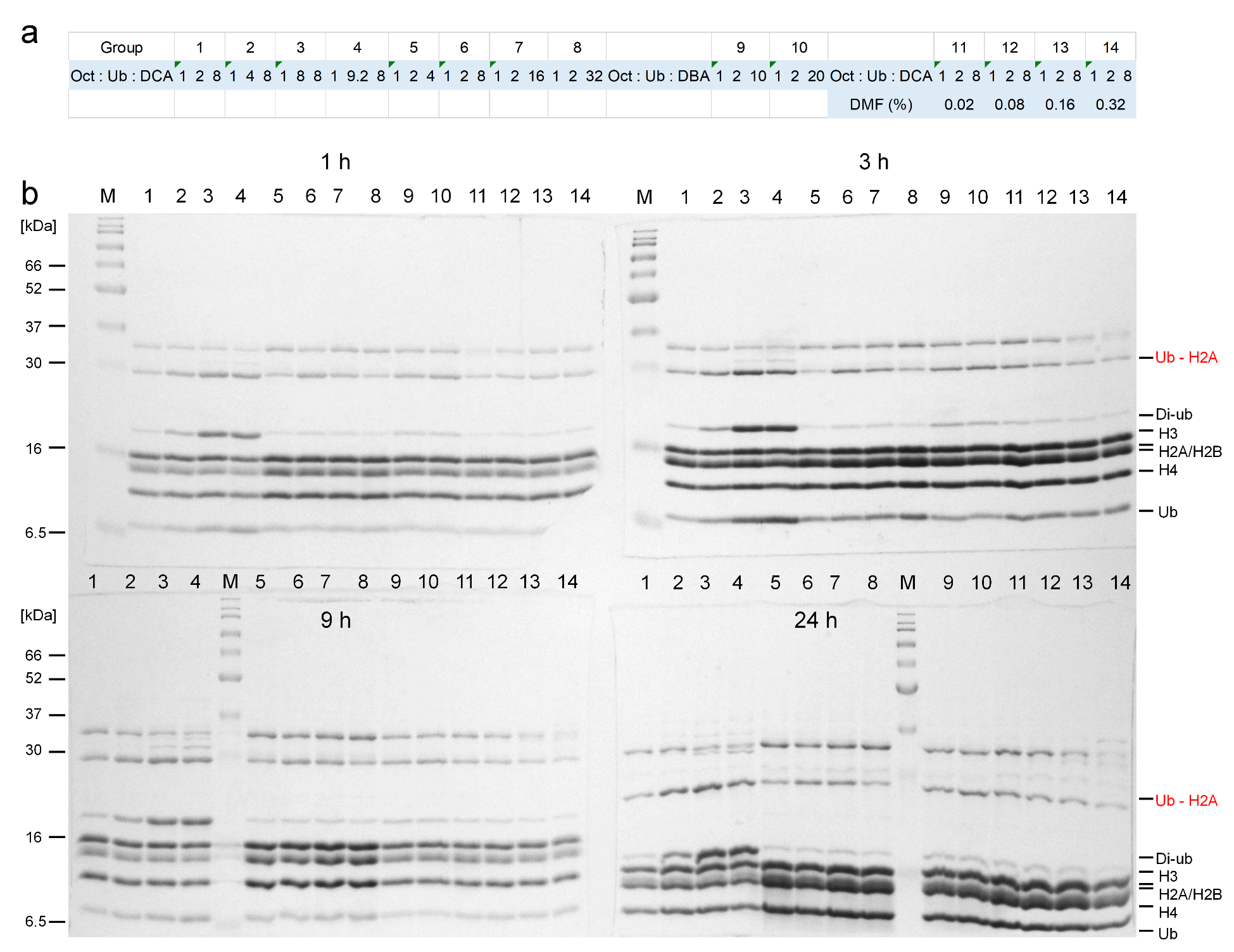


Figure S6. Supplementary materials for Fig. 2. (a) The table of crosslinking consists of the molar ratio of ubiquitin (G76C), 1,3-dichloroacetone, 1,3-dibromoacetone, and the percentage of DMF. (b) DCA/DBA-assisted crosslinking of ubiquitin (G76C) and histone octamer (H2AK119C) under different conditions (molar ratio of ubiquitin, DCA/DBA, precent of DMF, and different time point).


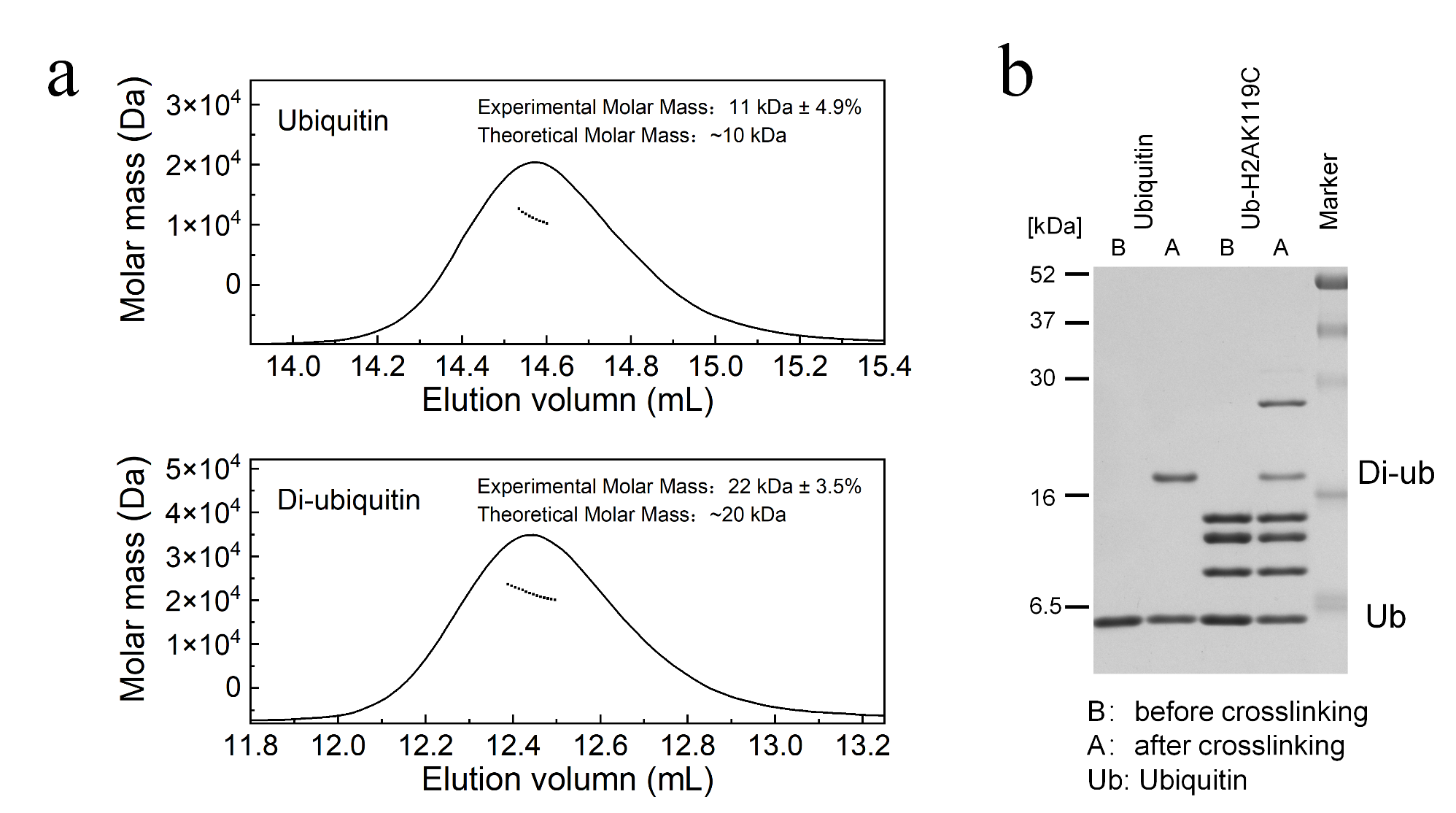


Figure S7. Supplementary materials for Fig. 3. a. The molecular weight of ubiquitin and di-ubiquitin before and after crosslinking was determined by SEC-MALS. The weight-average molar mass for ubiquitin and di-ubiquitin are plotted versus the elution volume, showing molar-mass values over the peak width. b. SDS-PAGE analysis of the ubiquitin and ub-H2AK119C before and after incubation with DBA.


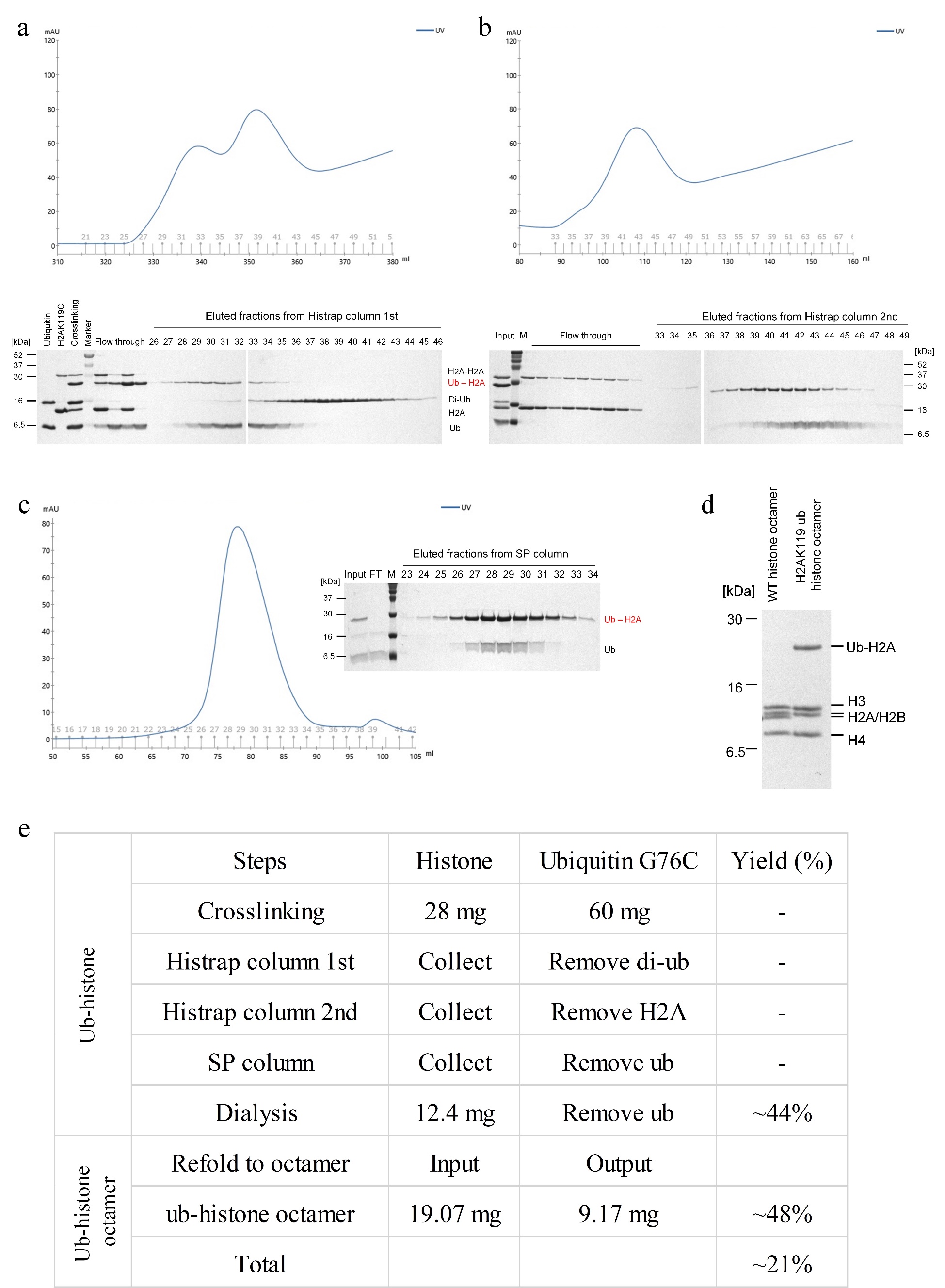


Figure S8. The synthesis and purification of the H2AK119ub histone by traditional methods. a. The crosslinking products of ubiquitin and H2AK119C histone were purified over Histrap column. The di-ubiquitin was almost removed in this step, and a portion of the target protein (ub-H2A) is present in the flow-through due to column saturation. b. The flow-through and the Peak1 from the first purification step were further purified over the Histrap column. c. The ub-histone was further purified over the SP column, and the majority of ubiquitin presented in the flow-through was removed. d. SDS-PAGE analysis of H2AK119ub histone octamers obtained through refolding methods. Residual ubiquitin was completely removed during dialysis step and histone octamer refolding step, the final H2AK119ub histone octamer is of high quality. e. The steps and yields for obtaining ubiquitinated histone octamers by traditional method.


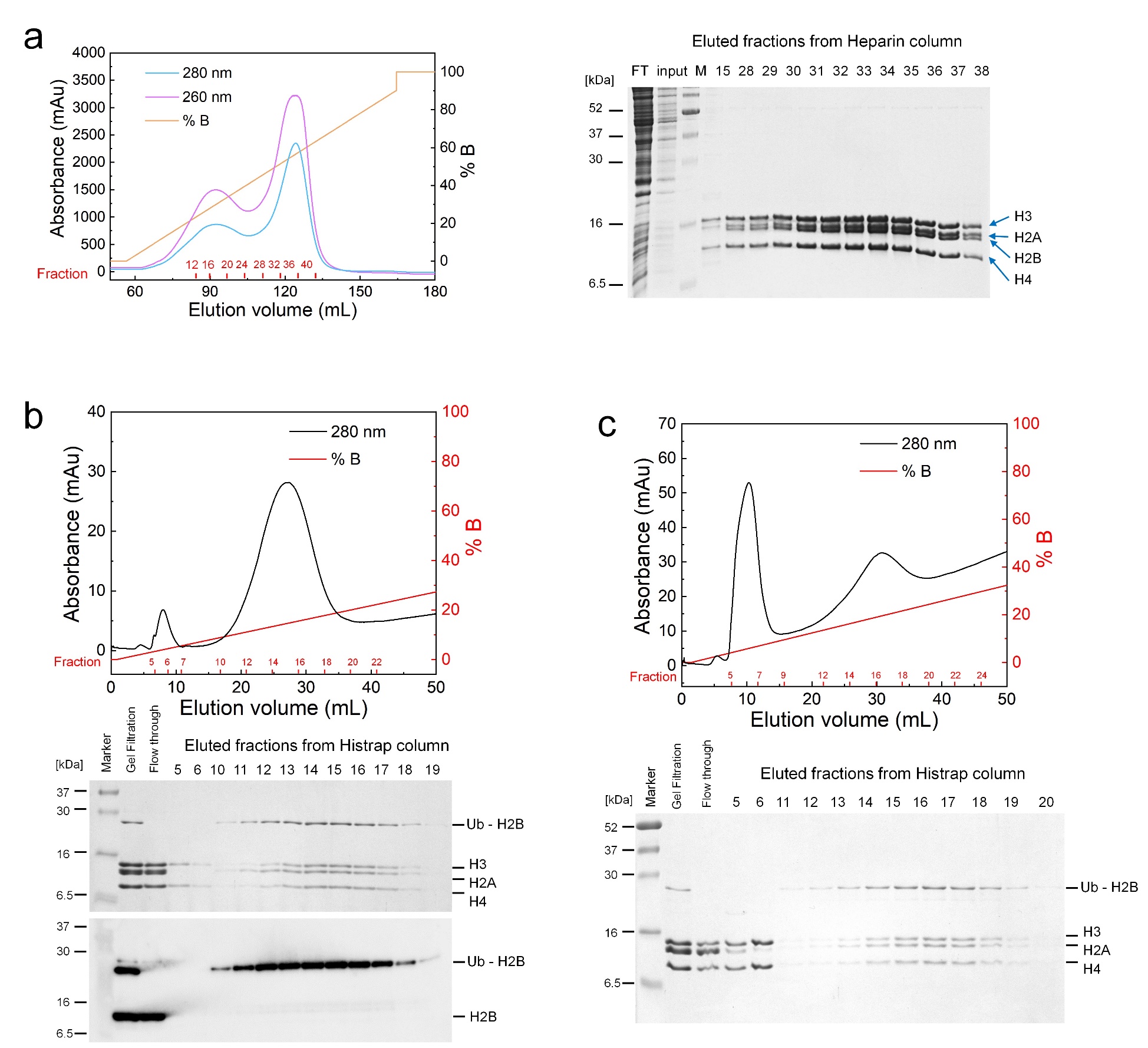


Figure S9. Supplementary materials for Fig. 3. (a) The H2BK120C histone octamer was purified overaffinity chromatography using a heparin column; (b) The purification results of H2BK120ub and (c) H2BK120ub-H3K79M histone octamer analogue.


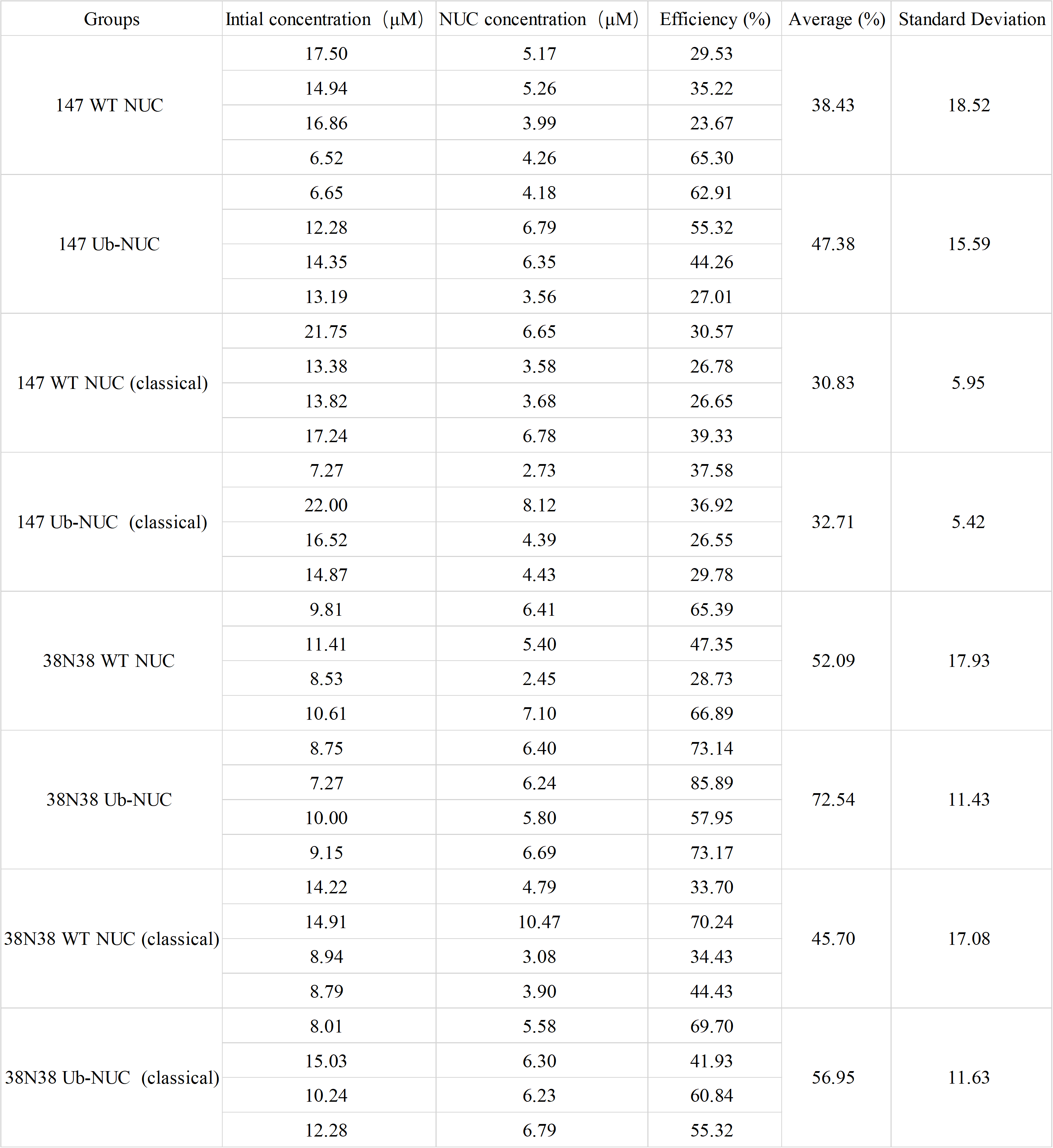


Figure S10. Supplementary materials for Fig. 4D. The table of efficiency of recovered nucleosomes by different methodologies.


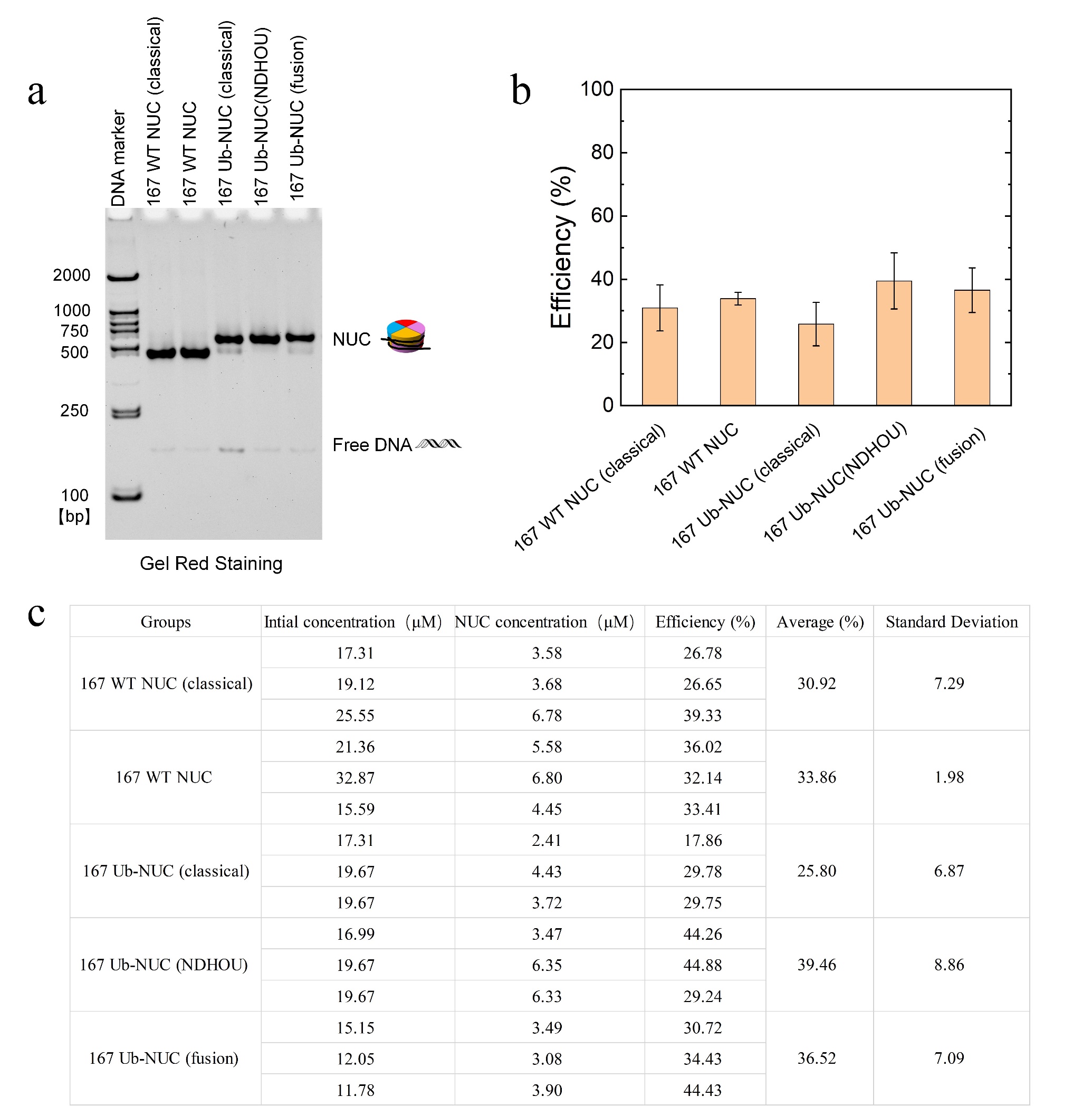


Figure S11. Supplementary materials for Fig. 4D. (a) Native-PAGE analysis of various 601-167 NCPs; WT NUC denotes unmodified nucleosomes, and Ub-NUC denotes ubiquitinated nucleosomes, with 'classical' referring to traditional reconstitution methods; (b) Comparison of nucleosome reconstitution efficiencies from different methodologies showing no significant differences, Error bars, s.d. (n = 3); (c) The table of yields of recovered nucleosomes by different methodologies.


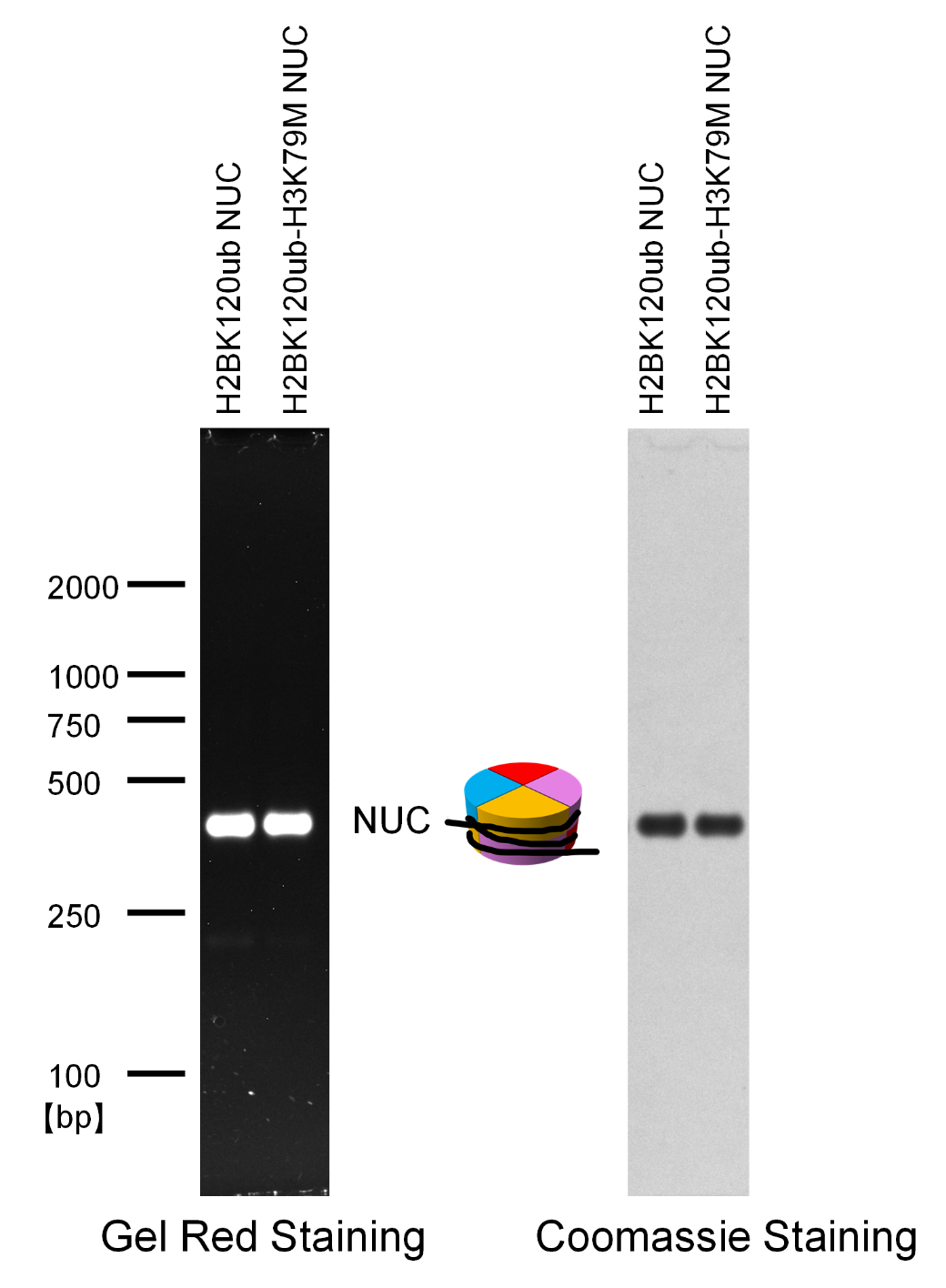


Figure S12. Supplementary materials for Fig. 4. The Native-PAGE gel analysis of NCPs reconstituted using H2BK120ub and H2BK120ub-H3K79M histone octamers with 147 bp double-stranded DNA by GelRed staining and Coomassie staining.


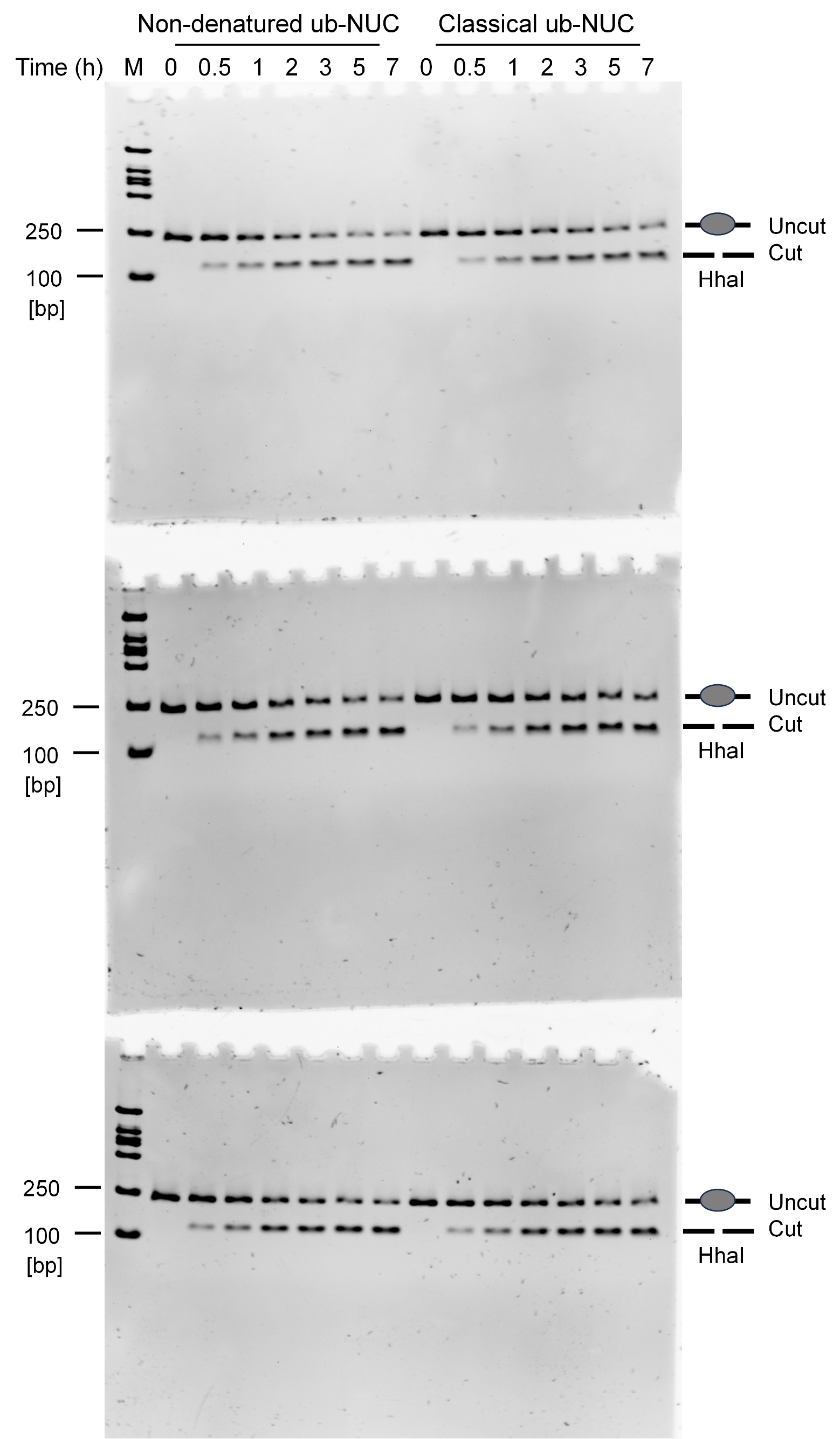


Figure S13: Supplementary materials for Fig. 5B. Original gels of the restriction enzyme accessibility assays of non-denatured and classical ubiquitinated nucleosomes under the ncBAF complex. The cut fractions were quantified from the three independent assays are shown in Fig. 5B.


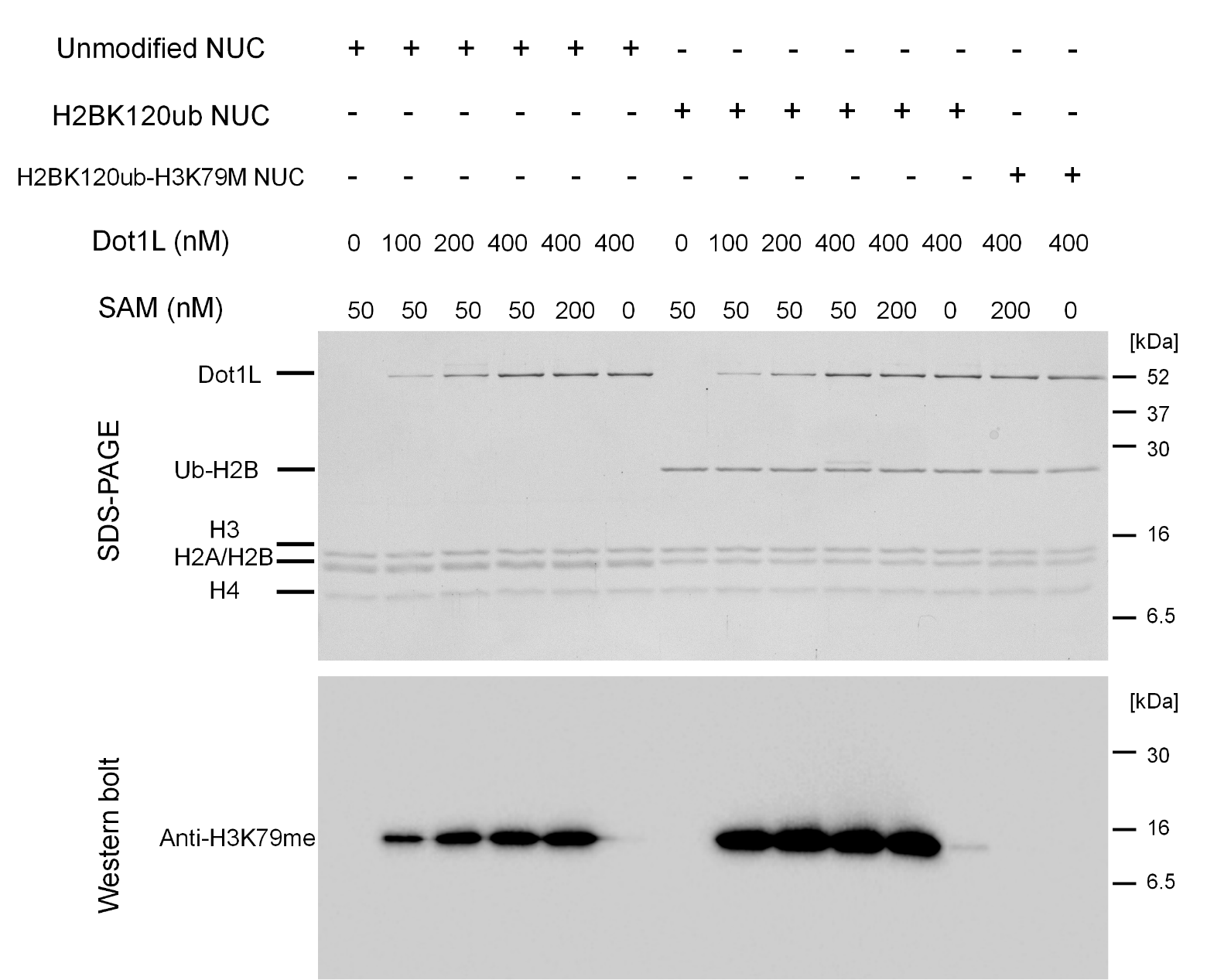


Figure S14. Supplementary materials for Fig. 5D. HMT assays with varying Dot1L (0, 100, 200, or 400 nM) or SAM (0, 50, or 200 nM) concentrations, in the presence of non-H2BK120ub nucleosomes (unmodified nucleosome), H2BK120ub nucleosomes and H2BK120ub-H3K79M nucleosomes. The results indicated that H2BK120 ubiquitinated nucleosomes from NDHOU approach undergo H3K79 methylation modification that is more intense compared to unmodified nucleosomes in the presence of Dot1L and SAM, with an enhanced H3K79 methylation observed as the concentration of Dot1L increases.


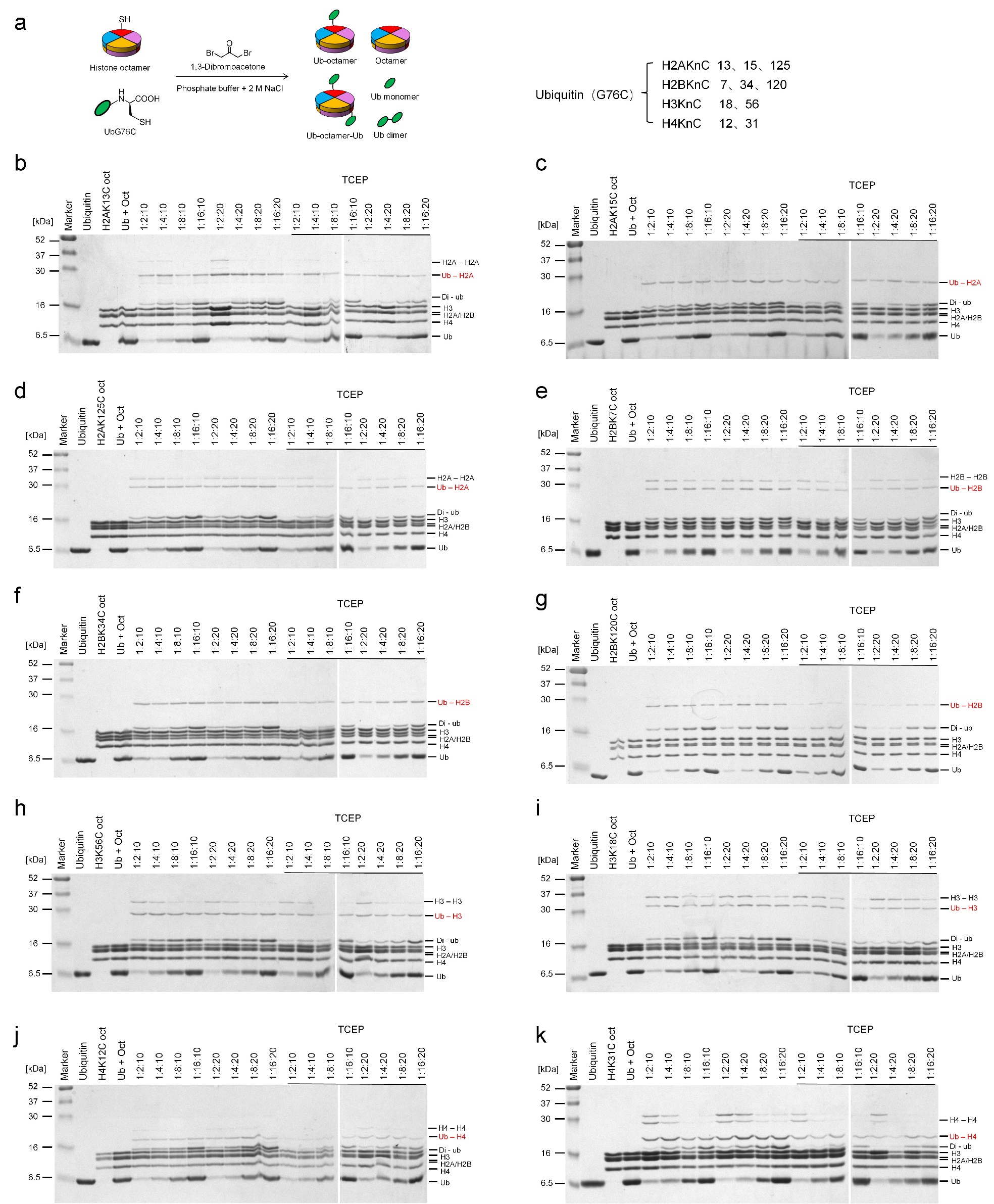


Figure S15. The universality of the DBA-assisted synthesis method was performed through several histone octamer mutants. (a) Schematic of DBA-assisted one-step synthesis method for ubiquitinated histone octamer mimic, including reaction conditions, potential products, and several histone octamer mutants. The SDS-PAGE analysis of the reaction products of histone octamer (b) H2AK13C (c) H2AK15C (d) H2AK125C (e) H2BK7C (f) H2BK34C (g) H2BK120C (h) H3K56C (i) H3K18C (j) H4K12C and (k) H4K31C, ubiquitin (G76C) and DBA after incubation on ice bath at the pH 7.5 for three hours at the molar ratio of 1:2:10, 1:4:10, 1:8:10, 1:16:10, 1:2:20, 1:4:20, 1:8:20, 1:16:20, and pretreatment with TCEP at the same time.


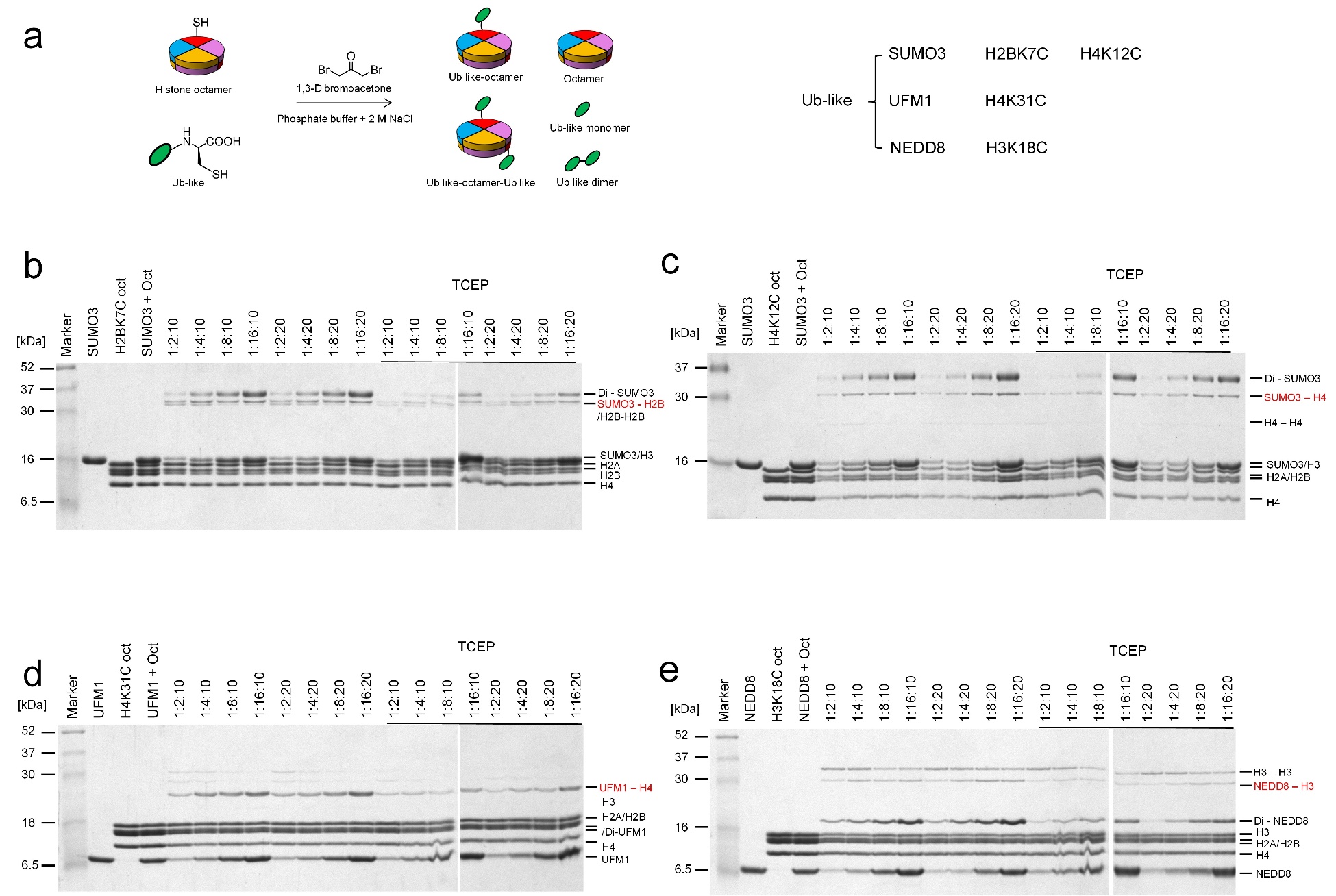


Figure S16. The universality of the DBA-assisted synthesis method was performed through three ubiquitin-like proteins (SUMO3, UFM1, and NEDD8). (a) Schematic of DBA-assisted one-step synthesis method for ubiquitin-like modification histone octamer mimic, including reaction conditions, potential products, and three ubiquitin-like proteins. The SDS-PAGE analysis of the reaction products of (b) H2BK7C and SUMO3 (c) H4K12C and SUMO3, (d) H4K31C and UFM1, (e) H3K18C and NEDD8 and DBA after incubation on ice bath at the pH 7.5 for three hours at the molar ratio of 1:2:10, 1:4:10, 1:8:10, 1:16:10, 1:2:20, 1:4:20, 1:8:20, 1:16:20, and pretreatment with TCEP at the same time.


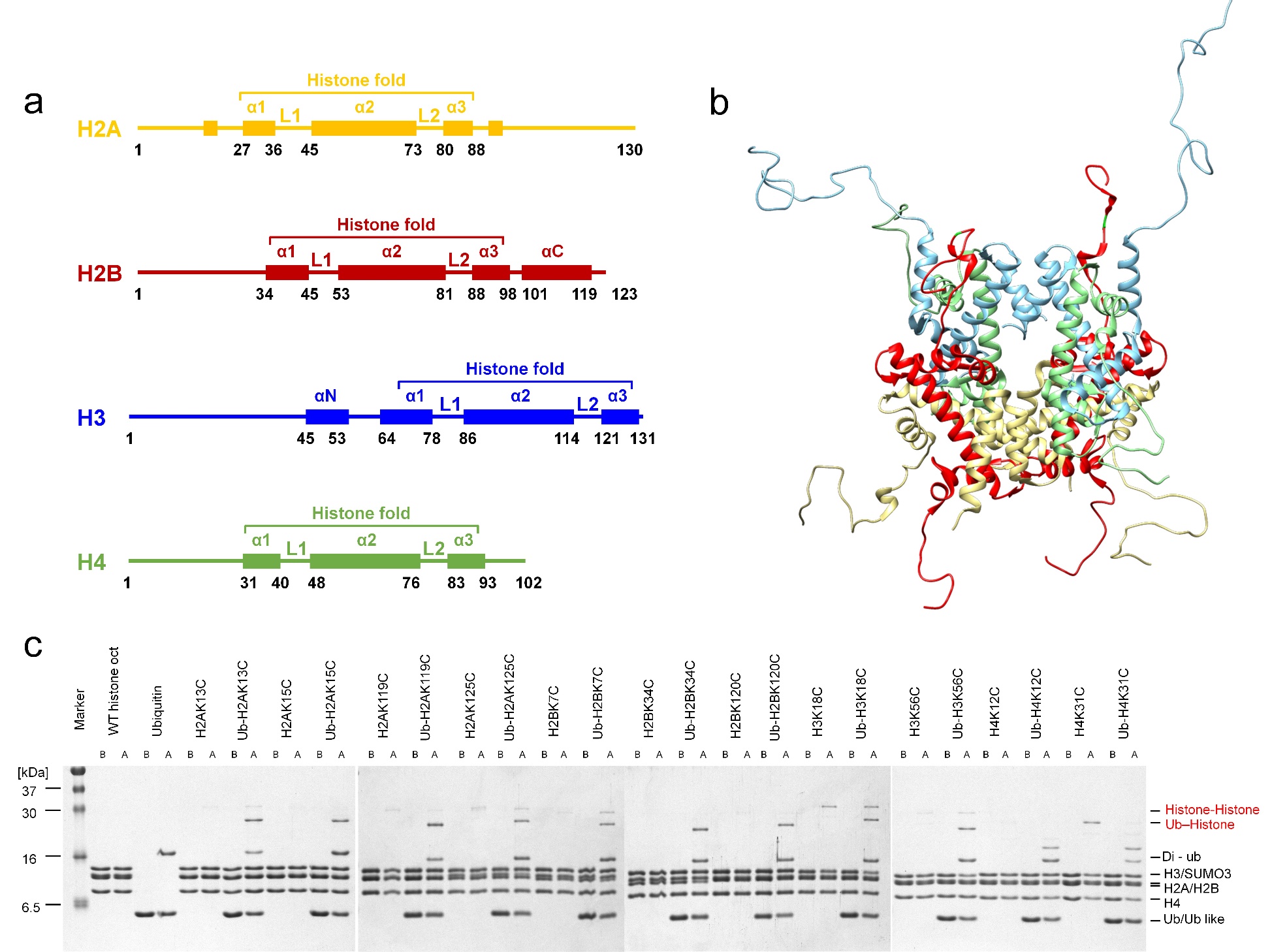


Figure S17. Supplementary materials for Fig. 6. (a) Domain organization of the four core histones. The intervening loops are shown as lines (labelled as L1 and L2), while the α-helices are depicted as boxes (labelled as α1, α2 and α3); (b) Nucleosome core particle structure (PDB ID 1KX5); (c) The SDS-PAGE gels showing the DBA-assisted crosslinking results of histone octamers self-crosslinking, histone octamers and ubiquitin or ubiquitin-like proteins, with 'B' indicating before and 'A' denoting after cross-linking.


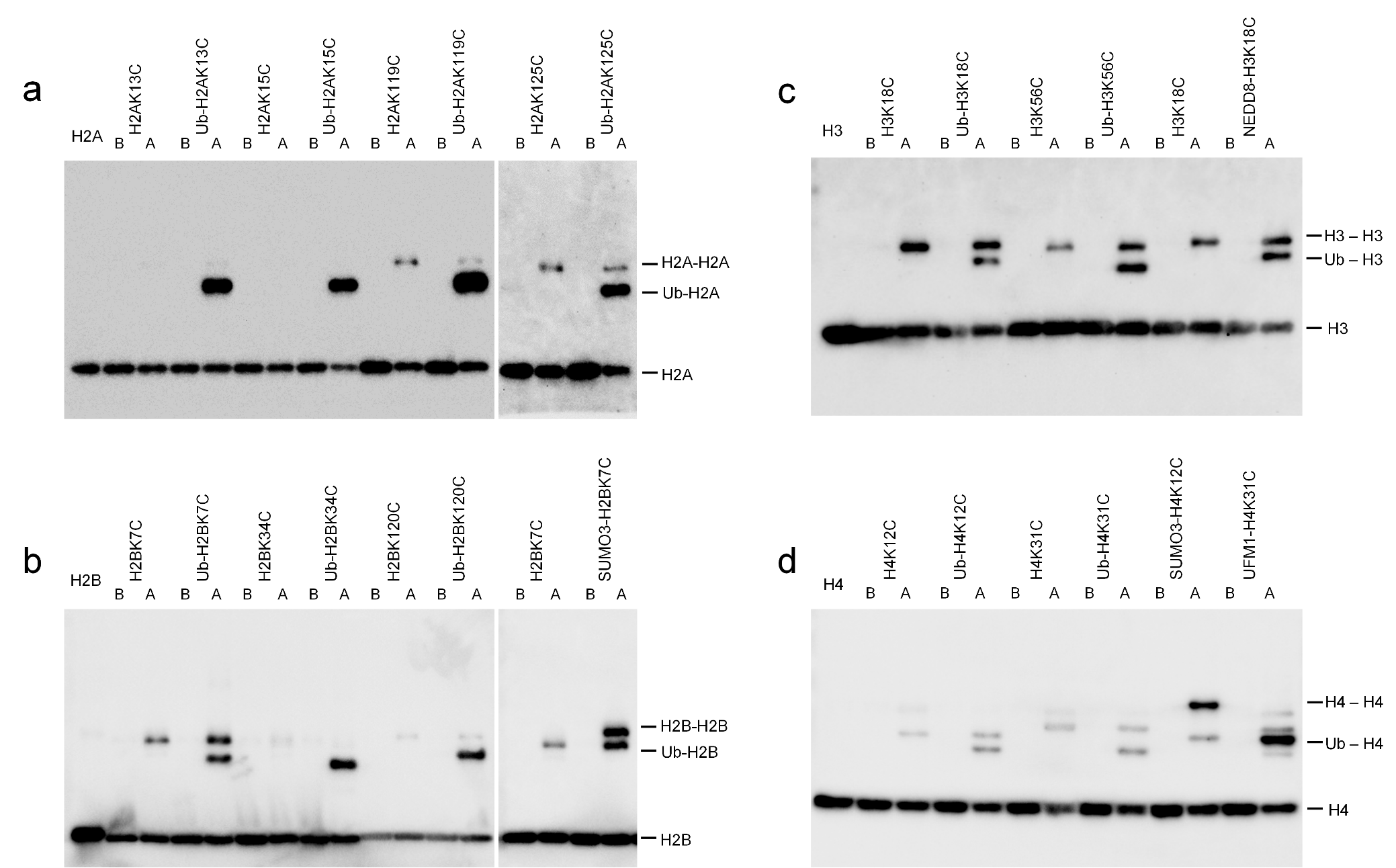


Figure S18. The Western blot showing the DBA-assisted crosslinking results of histone octamers self-crosslinking, histone octamers and ubiquitin or ubiquitin-like proteins, with 'B' indicating before and 'A' denoting after cross-linking.
